# Therapeutic Targeting of *MZF1‐AS1*/PARP1/E2F1 Axis Inhibits Proline Synthesis and Neuroblastoma Progression

**DOI:** 10.1002/advs.201900581

**Published:** 2019-08-10

**Authors:** Erhu Fang, Xiaojing Wang, Feng Yang, Anpei Hu, Jianqun Wang, Dan Li, Huajie Song, Mei Hong, Yanhua Guo, Yang Liu, Hongjun Li, Kai Huang, Liduan Zheng, Qiangsong Tong

**Affiliations:** ^1^ Department of Pediatric Surgery Union Hospital Tongji Medical College Huazhong University of Science and Technology 1277 Jiefang Avenue Wuhan 430022 Hubei Province P. R. China; ^2^ Clinical Center of Human Genomic Research Union Hospital Tongji Medical College Huazhong University of Science and Technology 1277 Jiefang Avenue Wuhan 430022 Hubei Province P. R. China; ^3^ Department of Pathology Union Hospital Tongji Medical College Huazhong University of Science and Technology 1277 Jiefang Avenue Wuhan 430022 Hubei Province P. R. China

**Keywords:** E2F transcription factor 1, myeloid zinc finger 1 antisense RNA 1, poly(ADP‐ribose) polymerase 1, proline synthesis, tumor progression

## Abstract

Proline synthesis plays an important role in the metabolic reprogramming that contributes to tumor progression. However, the mechanisms regulating expression of proline synthetic genes in neuroblastoma (NB) remain elusive. Herein, through integrative screening of a public dataset and amino acid profiling analysis, myeloid zinc finger 1 (*MZF1*) and *MZF1* antisense RNA 1 (*MZF1‐AS1*) are identified as transcriptional regulators of proline synthesis and NB progression. Mechanistically, transcription factor MZF1 promotes the expression of aldehyde dehydrogenase 18 family member A1 and pyrroline‐5‐carboxylate reductase 1, while proline facilitates the aggressiveness of NB cells. In addition, *MZF1‐AS1* binds poly(ADP‐ribose) polymerase 1 (PARP1) to facilitate its interaction with E2F transcription factor 1 (E2F1), resulting in transactivation of E2F1 and upregulation of *MZF1* and other oncogenic genes associated with tumor progression. Administration of a small peptide blocking *MZF1‐AS1*‐PARP1 interaction or lentivirus‐mediated short hairpin RNA targeting *MZF1‐AS1* suppresses the proline synthesis, tumorigenesis, and aggressiveness of NB cells. In clinical NB cases, high expression of *MZF1‐AS1*, *PARP1*, *E2F1*, or *MZF1* is associated with poor survival of patients. These results indicate that therapeutic targeting of *MZF1‐AS1*/PARP1/E2F1 axis inhibits proline synthesis and NB progression.

## 1 Introduction

Neuroblastoma (NB), a malignancy originating from neural crest cells, accounts for more than 15% of tumor‐related mortality in pediatric population.^1^ The outcome of advanced NB patients still remains poor, mainly due to tumor invasion and metastasis. Biosynthesis of proline, a nonessential amino acid (NEAA), is crucial for rapid proliferation and aggressiveness of tumor cells via activating mammalian target of rapamycin (mTOR)‐p70 ribosomal protein S6 kinase (p70S6K) pathway, resulting in phosphorylation and inactivation of eukaryotic translation initiation factor 4E binding protein 1 (4EBP1) and increase of protein synthesis.^2^, ^3^ Proline is produced via glutamate or ornithine route, which is mediated by sequential action of aldehyde dehydrogenase 18 family member A1 (ALDH18A1) or ornithine aminotransferase, and pyrroline‐5‐carboxylate reductase (PYCR).^3^ Meanwhile, proline can be converted to glutamate via proline catabolism regulated by proline dehydrogenase 1 (PRODH) and P5C dehydrogenase (P5CDH).^4^ Knockdown of *c‐MYC* decreases the expression of *ALDH18A1* and *PYCR1* and increases the expression of *PRODH*, resulting in reduced proline levels and survival of tumor cells.^5^ However, the mechanisms regulating proline metabolism in NB progression remain to be determined.

In recent years, emerging studies have shown the oncogenic or tumor suppressive roles of long noncoding RNAs (lncRNAs) in NB progression. For example, *lncUSMycN*, a lncRNA transcribed from upstream of *MYCN*, facilitates *MYCN* expression and proliferation of NB cells.^6^ LncRNA *MYCN* opposite strand (*MYCNOS*) cooperates with CCCTC‐binding factor to promote NB progression through increasing *MYCN* expression.^7^ In addition, E26 transformation‐specific sequence‐1 (*ETS1*) promoter‐associated noncoding RNA facilitates the proliferation and aggressiveness of NB cells via stabilizing β‐catenin.^8^ On the other hand, loss of tumor suppressive neuroblastoma associated transcript‐1 (*NBAT‐1*) contributes to NB progression by increasing proliferation and reducing differentiation of neuronal precursors.^9^ Meanwhile, the roles of lncRNAs in proline synthesis during NB progression still remain elusive.

In this study, we identify myeloid zinc finger 1 (MZF1) as a crucial transcription factor (TF) for proline synthesis and NB progression. *MZF1* antisense RNA 1 (*MZF1‐AS1*), an unstudied lncRNA, binds poly(ADP‐ribose) polymerase 1 (PARP1) to facilitate its interaction with E2F transcription factor 1 (E2F1), resulting in transactivation of E2F1 and upregulation of *MZF1* and other oncogenic genes. Preclinically, administration of a small peptide‐blocking *MZF1‐AS1*‐PARP1 interaction or lentivirus‐mediated short hairpin RNA (shRNA) targeting *MZF1‐AS1* significantly suppresses the proline synthesis, tumorigenesis, and aggressiveness, indicating the crucial roles of *MZF1‐AS1*/PARP1/E2F1 axis in tumor progression.

## 2 Results

### 2.1 *MZF1* Facilitates the Expression of Proline Synthetic Genes in NB

**Figure 1:**
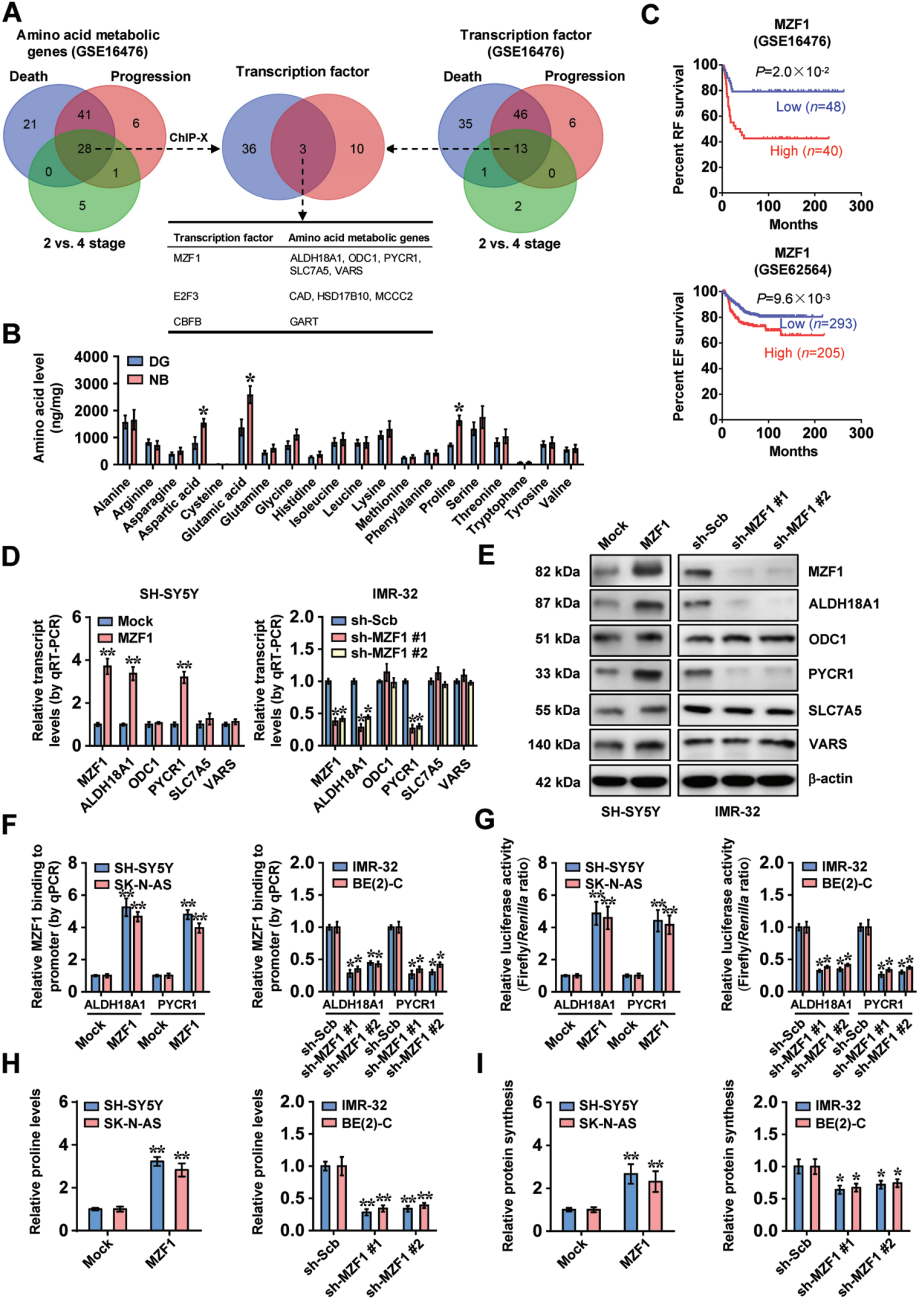
*MZF1* facilitates the expression of proline synthetic genes in NB. A) Venn diagram indicating amino acid metabolic genes (left panel) and transcription factors (right panel) differentially expressed (*P* < 0.05) in 88 NB cases (GSE16476) with various status of death, clinical progression, or INSS stages, and overlapping analysis with potential transcription factors regulating amino acid metabolic genes revealed by ChIP‐X program (middle panel). B) Ultra‐high performance liquid chromatography (UHPLC)‐mass spectrometry (MS)/MS analysis of amino acid contents in normal dorsal root ganglia (DG, *n* = 10) and NB tissues (*n* = 30). C) Kaplan–Meier curves showing the relapse‐free (RF) and event‐free (EF) survival of 88 (GSE16476) and 498 (GSE62564) NB cases with low or high levels of *MZF1* (cutoff values = 226.6 and 35.1). D,E) Real‐time quantitative reverse transcription‐polymerase chain reaction (qRT‐PCR), D) normalized to β‐actin, *n* = 4, and E) Western blot assays indicating the expression of *MZF1*, *ALDH18A1*, *ODC1*, *PYCR1*, *SLC7A5*, and *VARS* in SH‐SY5Y and IMR‐32 cells stably transfected with empty vector (mock), *MZF1*, scramble shRNA (sh‐Scb), sh‐MZF1 #1, or sh‐MZF1 #2. F,G) ChIP and quantitative polymerase chain reaction (qPCR), F) normalized to input, and G) dual‐luciferase assays showing the MZF1 enrichment and promoter activity of *ALDH18A1* and *PYCR1* in NB cells stably transfected with mock, *MZF1*, sh‐Scb, sh‐MZF1 #1, or sh‐MZF1 #2 (*n* = 4). H) Proline levels in NB cells stably transfected with mock, *MZF1*, sh‐Scb, sh‐MZF1 #1, or sh‐MZF1 #2 (*n* = 4). I) De novo protein synthesis in NB cells stably transfected with mock, *MZF1*, sh‐Scb, sh‐MZF1 #1, or sh‐MZF1 #2 (*n* = 4). Fisher's exact test for overlapping analysis in pane l (A). Student's *t*‐test and analysis of variance compared the difference in panels (B) and (D), and F–I). Log‐rank test for survival comparison in panel (C). *P < 0.05, ** *P* < 0.01 versus DG, mock, or sh‐Scb. Data are shown as mean ± s.e.m. (error bars) and representative of three independent experiments in panels (D)–(I).

To identify essential transcriptional regulator of amino acid metabolism in tumors, we performed comprehensive analysis of a public NB dataset of 88 cases (GSE16476),^10^ and identified 90, 76, and 34 amino acid metabolic genes differentially expressed (*P* < 0.05) in NB specimens with varied status of death, clinical progression, or international neuroblastoma staging system (INSS) stages (stage 2 vs 4), respectively (**Figure** **1**A). Based on overlapping analysis of these results (*P* < 0.001), 28 amino acid metabolic genes were found to be consistently associated with death, progression, and advanced INSS stages of NB (Figure 1A). Similarly, 13 transcription factors were consistently associated with these clinical features of 88 NB cases (Figure 1A), which were subjective to further overlapping analysis with 39 transcription factors regulating 28 amino acid metabolic genes analyzed by ChIP‐X program.^11^ The results revealed MZF1 as the top transcription factor ranking by number of potential target genes involved in proline synthesis, ornithine metabolism, amino acid transportation, and amino acid linking to transfer RNAs (tRNAs), including *ALDH18A1*, ornithine decarboxylase 1 (*ODC1*), *PYCR1*, solute carrier family 7 member 5 (*SLC7A5*), and valyl‐tRNA synthetase (*VARS*, Figure 1A). Notably, amino acid profiling indicated significantly higher levels of proline and two other NEAAs (aspartic acid and glutamic acid) in NB tissues (Figure 1B). Supplementation of them facilitated the in vitro growth of SH‐SY5Y and IMR‐32 cells in Dulbecco's modified Eagle's medium (DMEM) lacking NEAAs (Figure S1A, Supporting Information).

Log‐rank test of 88 (GSE16476) and 498 (GSE62564)^12^ NB cases indicated that patients with high *MZF1* expression had poorer survival (*P* = 2.0 × 10^−2^ and *P* = 9.6 × 10^−3^; Figure 1C). In NB cell lines SH‐SY5Y, SK‐N‐AS, IMR‐32, and BE(2)‐C, colon cancer SW480 cells,^13^ and cervical cancer SiHa cells^14^ (representing low or high *MZF1* levels), stable overexpression or knockdown of *MZF1*, respectively, increased and decreased the expression of *ALDH18A1* and *PYCR1* (Figure 1D,E; Figure S1B–F, Supporting Information), two genes essential for proline synthesis and tumor progression.^15^, ^16^ Meanwhile, the expression of *ODC1*, *SLC7A5*, and *VARS* was not affected by ectopic overexpression or silencing of *MZF1* (Figure 1D,E; Figure S1C,D, Supporting Information). In addition, the expression of *PYCR2*, *PYCR3*, *PRODH*, or *P5CDH*, other genes involved in proline synthesis and catabolism,^4^ was not affected by MZF1 (Figure S1G,H, Supporting Information). The MZF1 enrichment and promoter activity of *ALDH18A1* and *PYCR1* were increased and decreased by overexpression or knockdown of *MZF1* in NB cells, respectively (Figure 1F,G). Consistently, mining of public datasets revealed the association of *ALDH18A1* or *PYCR1* levels with poor survival of NB patients (Figure S2A, Supporting Information). Moreover, *MZF1* levels were positively correlated with those of *ALDH18A1* (*R* = 0.414, *P* = 6.1 × 10^−5^; *R* = 0.246, *P* = 2.7 × 10^−8^) and *PYCR1* (*R* = 0.527, *P* = 1.4 × 10^−7^; *R* = 0.227, *P* = 2.9 × 10^−7^) in NB tissues (Figure S2B, Supporting Information). Importantly, overexpression of *MZF1* increased the ^13^C glutamine‐to‐proline conversion and proline levels in SH‐SY5Y and SK‐N‐AS cells, while knockdown of *MZF1* significantly attenuated their levels in IMR‐32 and BE(2)‐C cells (Figure 1H; Figure S2C, Supporting Information). Accordingly, ectopic expression or knockdown of *MZF1* increased and decreased the phosphorylation of mTOR/p70S6K and 4EBP1, resulting in increased and reduced de novo protein synthesis in NB cells, respectively (Figure 1I; Figure S2D, Supporting Information). These findings indicated that transcription factor MZF1 facilitated the expression of proline synthetic genes in NB.

### 2.2 *MZF1‐AS1* Promotes Proline Synthesis and NB Progression

**Figure 2:**
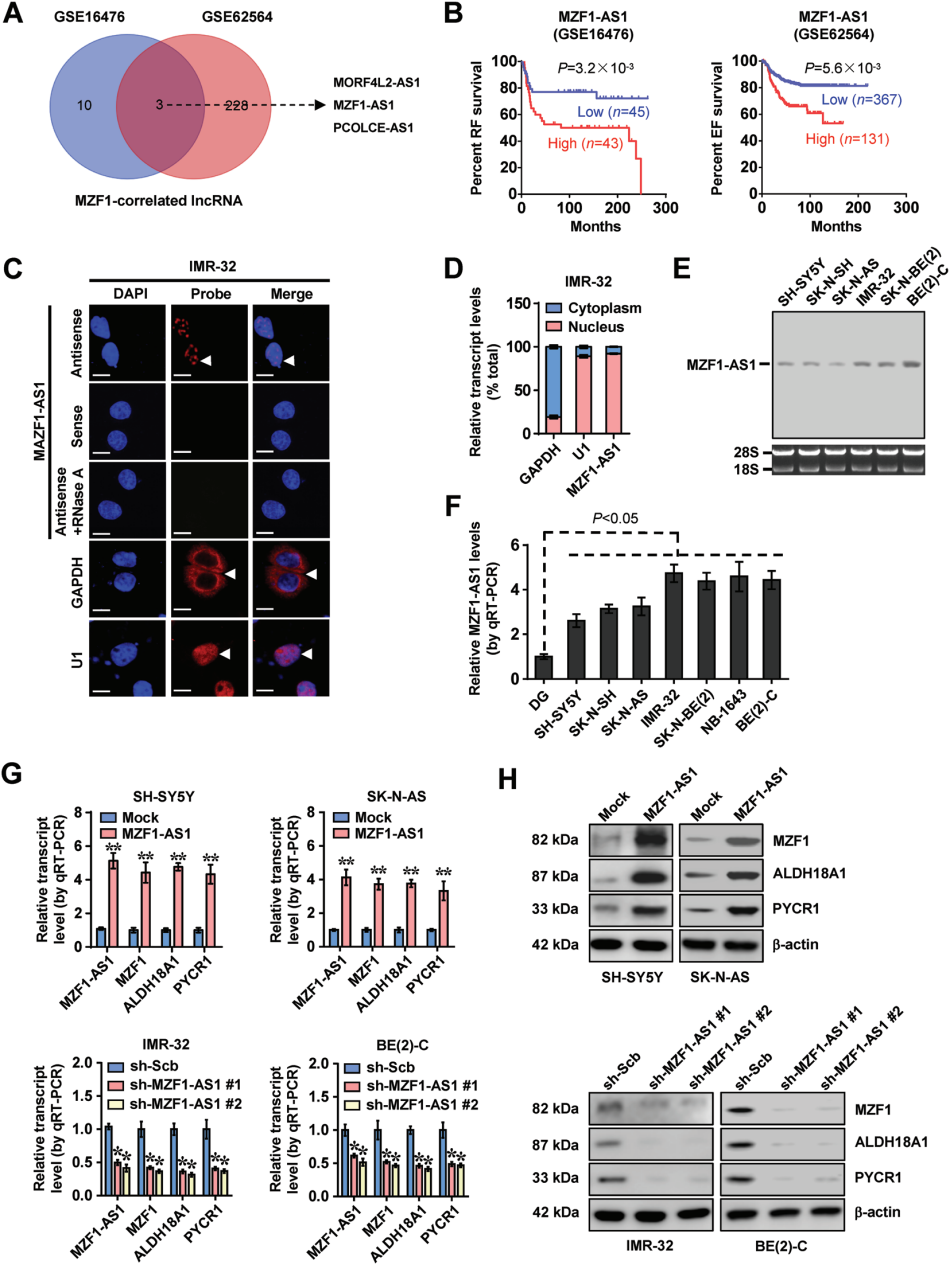
*MZF1‐AS1* facilitates the expression of *MZF1* and downstream proline synthetic genes. A) Venn diagram showing comprehensive analysis of *MZF1*‐correlated lncRNAs (*P* < 0.01) in 88 (GSE16476) and 498 (GSE62564) NB cases. B) Kaplan–Meier curves indicating the relapse‐free (RF) and event‐free (EF) survival of 88 (GSE16476) and 498 (GSE62564) NB cases with low or high levels of *MZF1‐AS1* (cutoff values = 9.2 and 7.3). C) RNA‐fluorescence in situ hybridization (FISH) using a 185‐bp antisense probe (red) revealing the localization (arrowheads) of *MZF1‐AS1* in IMR‐32 cells. Sense probe and RNase A (20 µg) treatment were used as negative controls, while glyceraldehyde 3‐phosphate dehydrogenase *(GAPDH)* and *U1* were applied as cytoplasmic and nuclear controls, with nuclei staining by 4′,6‐diamidino‐2‐phenylindole (DAPI, blue). Scale bars: 10 µm. D) Real‐time qRT‐PCR (normalized to β‐actin, *n* = 4) showing the enrichment of *MZF1‐AS1* in the cytoplasm and nuclei of IMR‐32 cells. E) Northern blot indicating the endogenous existence of *MZF1‐AS1* in cultured NB cells. F) Real‐time qRT‐PCR (normalized to β‐actin, *n* = 4) showing the expression of *MZF1‐AS1* in normal dorsal root ganglia (DG) and NB cell lines. G,H) Real‐time qRT‐PCR, G) normalized to β‐actin, *n* = 5, and H) Western blotassays revealing the expression of *MZF‐AS1*, *MZF1*, *ALDH18A1*, and *PYCR1* in NB cells stably transfected with empty vector (mock), *MZF1‐AS1*, scramble shRNA (sh‐Scb), or sh‐MZF1‐AS1. Fisher's exact test for overlapping analysis in panel (A). Log‐rank test for survival comparison in B. Student's *t*‐test and analysis of variance compared the difference in panels (F) and (G). *P < 0.05, ** *P* < 0.01 versus mock or sh‐Scb. Data are shown as mean ± s.e.m. (error bars) and representative of three independent experiments in panels (C)–(H).

To further investigate the lncRNA regulating *MZF1* expression in NB, mining of public datasets (GSE16476 and GSE62564) revealed that MORF4L2 antisense RNA 1 (*MORF4L2‐AS1*), *MZF1‐AS1*, and PCOLCE antisense RNA 1 (*PCOLCE‐AS1*) were associated with *MZF1* levels (**Figure** **2**A). Among them, only *MZF1‐AS1*, consisting of six exons and locating at chromosome 19q13.43, was consistently associated with poor survival of NB and other types of cancers (Figure 2B; Figure S3A, Supporting Information). The codon substitution frequency analysis^17^ revealed a low coding capacity of *MZF1‐AS1* (score = −79.446). In public datasets (GSE16476 and GSE62564), *MZF1‐AS1* expression was positively correlated with *MZF1* levels (*R* = 0.395, *P* = 1.4 × 10^−4^; *R* = 0.662, *P* = 2.3 × 10^−64^; Figure S3B, Supporting Information), and higher in NB tissues with death (*P* = 1.0 × 10^−2^), clinical progression (*P* = 3.9 × 10^−3^), high risk (*P* = 2.5 × 10^−3^), or unfavorable histology (*P* = 5.2 × 10^−3^; Figure S3C, Supporting Information). The 2600 nt *MZF1‐AS1* was abundant in NB cells, mainly localizing in the nucleus (Figure 2C–E; Figure S4A, Supporting Information). However, ectopic expression or knockdown of *MZF1* did not affect the *MZF1‐AS1* levels of NB cells (Figure S4B, Supporting Information).

**Figure 3:**
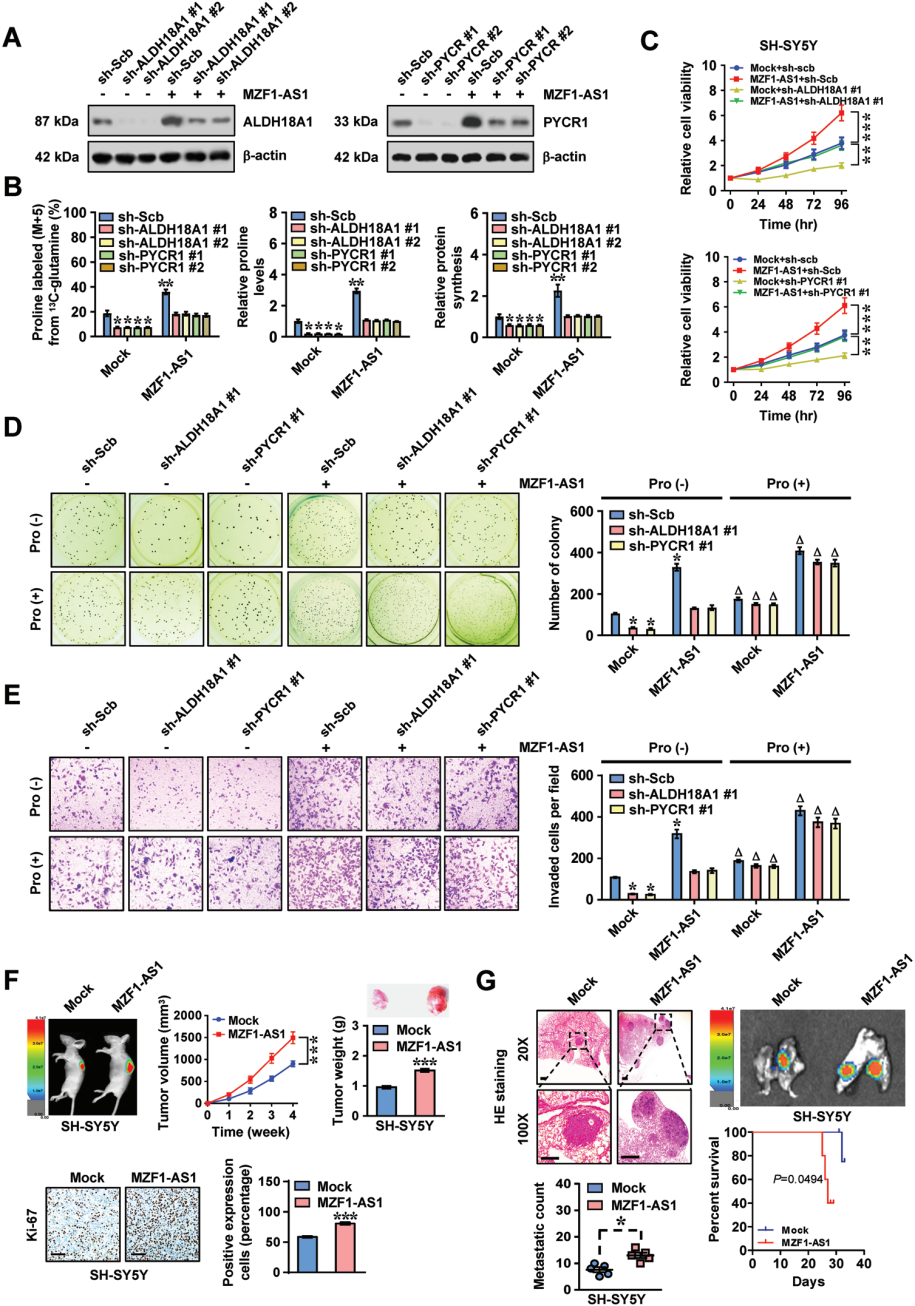
***MZF1‐AS1*** promotes proline synthesis and NB progression. A) Western blot assay indicating the levels of ALDH18A1 or PYCR1 in SH‐SY5Y cells stably transfected with empty vector (mock) or *MZF1‐AS1*, and those co‐transfected with scramble shRNA (sh‐Scb), sh‐ALDH18A1, or sh‐PYCR1. B) ^13^C glutamine‐to‐proline conversion (left panel), proline levels (middle panel), and de novo protein synthesis (right panel) in NB cells stably transfected with mock or *MZF1‐AS1*, and those co‐transfected with sh‐Scb, sh‐ALDH18A1, or sh‐PYCR1. C–E) MTT C) colorimetric, representative images (left panel) and quantification (right panel) of D) soft agar and E) matrigel invasion assays showing the viability, anchorage‐independent growth, and invasion of SH‐SY5Y cells stably transfected with mock or *MZF1‐AS1*, and those co‐transfected with sh‐Scb, sh‐ALDH18A1 #1, or sh‐PYCR1 #1, with or without proline (Pro, 3 × 10^−3^ m) supplementation (*n* = 4). F) Representative images (left upper panel), in vivo growth curve (middle upper panel), weight at the end points (right upper panel), representative images (left lower panel) and quantification (right lower panel) of Ki‐67 expression of xenograft tumors formed by subcutaneous injection of SH‐SY5Y cells stably transfected with mock or *MZF1‐AS1* into dorsal flanks of nude mice (*n* = 5 per group). G) Hematoxylin and eosin (H&E) staining (left upper panel), representative images (right upper panel), metastatic counts of lungs (left lower panel), and Kaplan–Meier curves (right lower panel) of nude mice (*n* = 5 per group) treated with tail vein injection of SH‐SY5Y cells stably transfected with mock or *MZF1‐AS1*. Scale bar: 100 µm. Student's *t*‐test and analysis of variance compared difference in panels (B)–(G). Log‐rank test for survival comparison in panel (G). *P < 0.05, ** *P* < 0.01, *** *P* < 0.001 versus mock+sh‐Scb or mock. ^Δ^ *P* < 0.05 versus Pro (‐). Data are shown as mean ± s.e.m. (error bars) and representative of three independent experiments in panels (A)–(E).

In SH‐SY5Y, SK‐N‐AS, IMR‐32, and BE(2)‐C cells (representing low or high *MZF1‐AS1* levels), stable overexpression or knockdown of *MZF1‐AS1* led to significantly increased and decreased expression of *MZF1* and its downstream genes *ALDH18A1* and *PYCR1*, respectively (Figure 2F–H). In the absence of exogenous proline, silencing of *ALDH18A1* or *PYCR1* prevented the SH‐SY5Y cells from increase of ^13^C glutamine‐to‐proline conversion, proline levels, protein synthesis, cell viability, anchorage‐independent growth, and invasiveness induced by stable overexpression of *MZF1‐AS1* (**Figure** **3**A–E). Supplementation of proline restored, for the most part, the decrease of growth and invasiveness induced by knockdown of *ALDH18A1* or *PYCR1* in SH‐SY5Y cells stably overexpressing *MZF1‐AS1* (Figure 3D,E). Stable overexpression of *MZF1‐AS1* into SH‐SY5Y cells led to a significant increase in growth, tumor weight, and Ki‐67 proliferative index of subcutaneous xenograft tumors (Figure 3F), and more lung metastatic counts and less survival of nude mice (Figure 3G). In contrast, stable silencing of *MZF1‐AS1* in IMR‐32 and BE(2)‐C cells led to reduction of ^13^C glutamine‐to‐proline conversion, proline levels, protein synthesis, cell viability, anchorage‐independent growth, and invasiveness in vitro (Figure S4C–F, Supporting Information), and significantly decreased the growth, tumor weight, and Ki‐67 proliferative index of xenograft tumors in nude mice (Figure S4G, Supporting Information). Less lung metastatic counts and higher survival possibility were observed in nude mice receiving tail vein injection of IMR‐32 cells with stable *MZF1‐AS1* knockdown (Figure S4H, Supporting Information). Together, these data indicated the oncogenic roles of *MZF1‐AS1* in proline metabolism and NB progression.

### 2.3 *MZF1‐AS1* Physically Interacts with PARP1 in NB Cells

**Figure 4:**
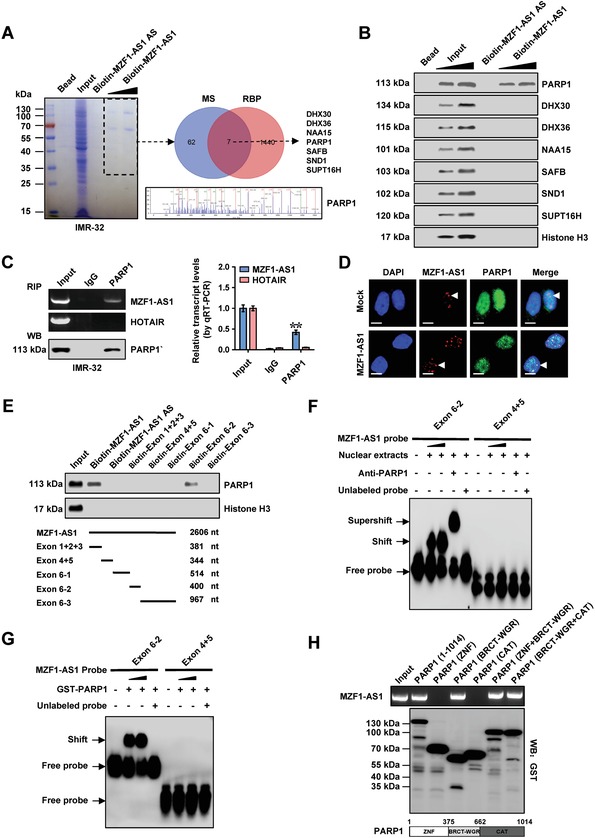
*MZF1‐AS1* physically interacts with PARP1 in NB cells. A) Mass spectrometry (MS) assay of indicated electrophoretic bands in Coomassie blue staining (left panel) and Venn diagram (right panel) indicating differential proteins pulled down by biotin‐labeled *MZF1‐AS1* from nuclear extracts of IMR‐32 cells, and overlapping analysis with RBP. B) Biotin‐labeled RNA pull‐down and Western blot assays showing protein pulled down by *MZF1‐AS1* from lysates of IMR‐32 cells. The *MZF1‐AS1* antisense (AS) and bead‐bound protein served as negative controls. C) RIP (left panel) and real‐time qRT‐PCR (right panel, normalized to input, *n* = 4) assays using PARP1 antibody indicating the interaction between *MZF1‐AS1* and PARP1 in IMR‐32 cells. The immunoglobulin G (IgG) and *HOTAIR* were applied as negative controls. D) Confocal images showing the co‐localization of *MZF1‐AS1* and PARP1 protein in the nuclei of IMR‐32 cells stably transfected with empty vector (mock) or *MZF1‐AS1*. Scale bars: 10 µm. E) Biotin‐labeled RNA pull‐down assay (upper panel) revealing the interaction between *MZF1‐AS1* truncations (lower panel) and PARP1 protein in SH‐SY5Y cells. Biotin‐labeled AS *MZF1‐AS1* served as a negative control. F,G) RNA electrophoretic mobility shift assay (EMSA) assay using biotin‐labeled probes (corresponding to 1240–1639 nt) indicating the interaction of *MZF1‐AS1* with endogenous PARP1. F) within nuclear extracts of SH‐SY5Y cells or G) glutathione S‐transferase (GST)‐tagged recombinant PARP1 protein, with or without treatment using PARP1 antibody or competition using an excess of unlabeled homologous probe. H) In vitro binding assay showing the recovered *MZF1‐AS1* levels detected by RT‐PCR (upper panel) after incubation with full‐length or truncation forms of GST‐tagged recombinant PARP1 protein validated by Western blot (lower panel). Fisher's exact test for overlapping analysis in A. Student's *t*‐test compared the difference in panel ©. ** *P* < 0.01 versus IgG. Data are shown as mean ± s.e.m. (error bars) and representative of three independent experiments in panels (B)–(H).

To further functionally characterize *MZF1‐AS1*, we screened potential *MZF1‐AS1*‐binding proteins via biotin‐labeled RNA pull‐down followed by mass spectrometry, which revealed 69 proteins pulled down by *MZF1‐AS1* in IMR‐32 cells (**Figure** **4**A; Table S1, Supporting Information). Overlapping analysis with RNA‐binding proteins (RBPs) defined by RBPDB (http://rbpdb.ccbr.utoronto.ca) revealed seven potential partners of *MZF1‐AS1* (Figure 4A), including DExH‐box helicase 30 (DHX30), DEAH‐box helicase 36 (DHX36), *N*(α)‐acetyltransferase 15 (NAA15), PARP1, scaffold attachment factor B (SAFB), staphylococcal nuclease, and tudor domain containing 1 (SND1), and FACT component suppressor of Ty homolog‐16 (SUPT16H). Further validating biotin‐labeled RNA pull‐down and Western blot assays demonstrated the physical interaction of *MZF1‐AS1* with PARP1, but not with DHX30, DHX36, NAA15, SAFB, SND1, or SUPT16H (Figure 4B). RNA immunoprecipitation (RIP) assay confirmed the specific binding of PARP1 to *MZF1‐AS1*, but not to control lncRNA HOX transcript antisense RNA (*HOTAIR*), in IMR‐32 cells (Figure 4C). Ectopic expression of *MZF1‐AS1* increased the nuclear co‐localization of *MZF1‐AS1* and PARP1 in IMR‐32 cells (Figure 4D). Exon 6 of *MZF1‐AS1*, especially the region of 1240–1639 nt, was responsible for its interaction with PARP1 protein (Figure 4E–G). The BRCA1 C‐terminus (BRCT)–tryptophan–glycine– arginine‐rich (WGR; 376–662 amino acids (aa)), but not zinc finger (ZNF; 1–375 aa) or catalytic (CAT; 663–1014 aa) domain, of glutathione S‐transferase (GST)‐tagged PARP1 protein was crucial for its interaction with *MZF1‐AS1* (Figure 4H). These results indicated that *MZF1‐AS1* physically interacted with PARP1 protein in NB cells.

### 2.4 *MZF1‐AS1* Increases E2F1 Target Gene Expression Through PARP1

**Figure 5:**
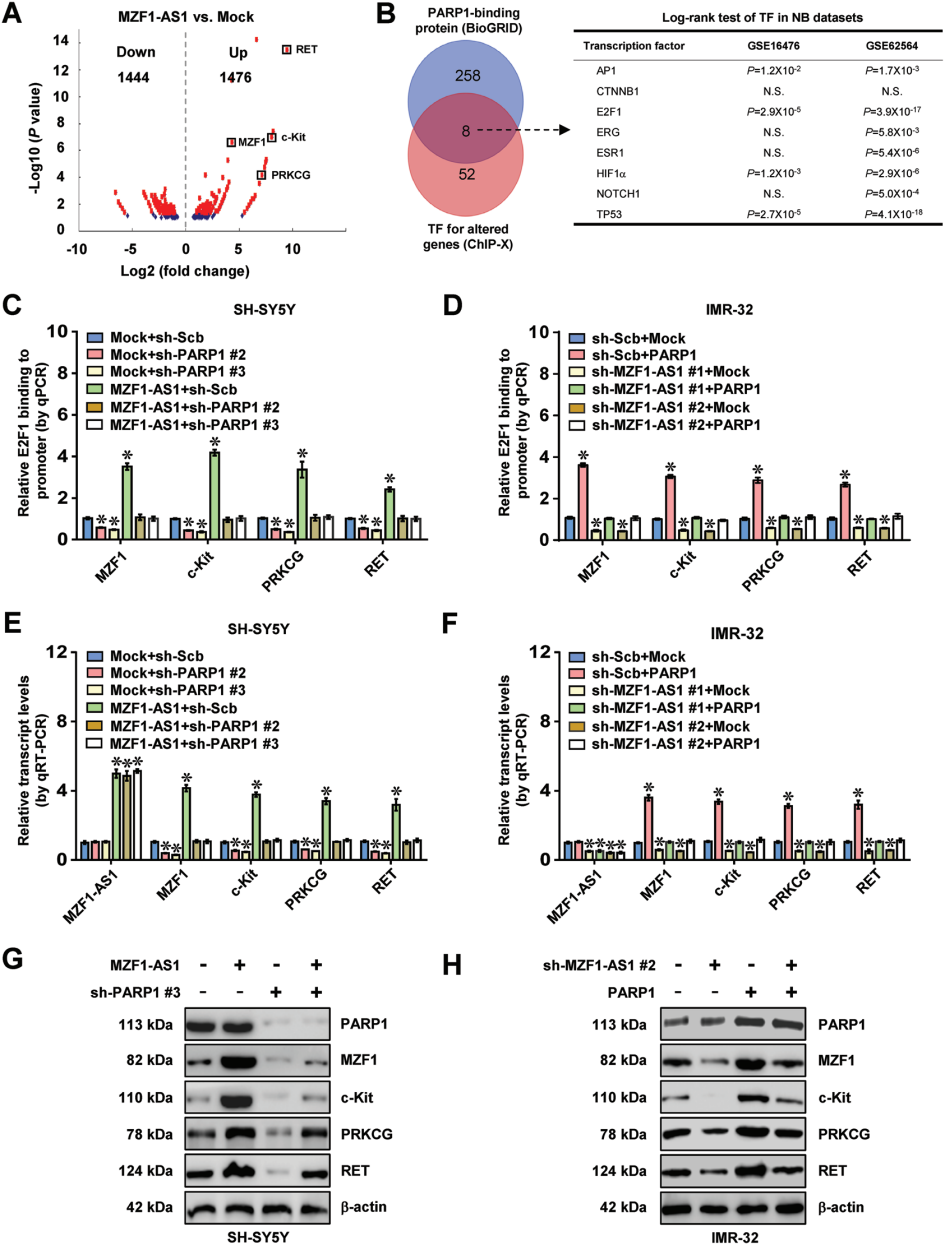
*MZF1‐AS1* increases E2F1 target gene expression through PARP1. A) Volcano plots of RNA‐seq revealing the alteration of gene expression (fold change >1.5, *P* < 0.05) in SH‐SY5Y cells stably transfected with empty vector (mock) or *MZF1‐AS1*. B) Venn diagram (left panel) showing identification of transcription factors (TFs) regulating target gene expression, based on overlapping analysis of potential TFs using ChIP‐X program and PARP1‐interacting proteins from BioGRID database. Log‐rank test (right panel) further revealing the association of identified TFs with overall survival of 88 (GSE16476) and 498 (GSE62564) NB cases. N.S., nonsignificant. C–F) ChIP and qPCR, C,D) normalized to input, and real‐time qRT‐PCR, E,F) normalized to β‐actin, assays indicating the E2F1 enrichment and transcript levels of target genes in SH‐SY5Y and IMR‐32 cells stably transfected with mock, *MZF1‐AS1*, scramble shRNA (sh‐Scb), or sh‐MZF1‐AS1, and those co‐transfected with sh‐PARP1 or *PARP1* (*n* = 5). G,H) Western blot showing the levels of target genes in SH‐SY5Y and IMR‐32 cells stably transfected with mock, *MZF1‐AS1*, sh‐Scb, or sh‐MZF1‐AS1 #2, and those co‐transfected with sh‐PARP1 #3 or *PARP1*. Fisher's exact test for overlapping analysis in panel (B). Log‐rank test for survival comparison in panel (B). Student's *t*‐test and analysis of variance compared difference in panels (C)–(F). * *P* < 0.05 versus mock+sh‐Scb. Data are shown as mean ± s.e.m. (error bars) and representative of three independent experiments in panels (C)–(H).

To identify the putative targets of *MZF1‐AS1*, RNA sequencing (RNA‐seq) assay revealed 1476 upregulated and 1444 downregulated genes (fold change > 1.5, *P* < 0.05) in SH‐SY5Y cells upon *MZF1‐AS1* overexpression (**Figure** **5**A). Further overlapping analysis of transcriptional regulators of these altered genes by ChIP‐X program and PARP1‐interacting protein in BioGRID database^18^ revealed eight potential transcription factors (Figure 5B). Among them, the expression of activator protein 1 (*AP1*), *E2F1*, hypoxia inducible factor 1 subunit alpha (*HIF1α*), or *TP53* was significantly associated with survival of NB patients (GSE16476 and GSE62564, Figure 5B). Of note, stable overexpression or knockdown of *MZF1‐AS1* altered the activity of E2F1, but not of AP1, HIF1α, or TP53, in SH‐SY5Y and IMR‐32 cells (Figure S5A,B, Supporting Information). Among 90 E2F1 target genes derived from RNA‐seq results and ChIP‐X analysis, the expression of *MZF1*, *c‐Kit*, protein kinase C gamma (*PRKCG*), or *RET* was most significantly altered (Figure 5A). In starved NB cells treated with serum stimulation, the expression profile of *MZF1* and its downstream genes *ALDH18A1* and *PYCR1*, but not of *c‐Kit*, *PRKCG*, or *RET*, was similar to that of *E2F1* around G1‐to‐S‐phase transition (Figure S5C, Supporting Information). Stable overexpression or silencing of *MZF1‐AS1* increased and decreased the E2F1 enrichment on target gene promoters in SH‐SY5Y and IMR‐32 cells, which were rescued by knockdown or ectopic expression of *PARP1*, respectively (Figure 5C,D). Meanwhile, knockdown or ectopic expression of *PARP1* or *E2F1* prevented NB cells from alteration in expression of *MZF1*, *c‐Kit*, *PRKCG*, and *RET* induced by overexpression or silencing of *MZF1‐AS1* (Figure 5E–H; Figure S5D–I, Supporting Information). These findings suggested that *MZF1‐AS1* increased the expression of E2F1 target genes through PARP1 in NB cells.

### 2.5 *MZF1‐AS1* Facilitates Proline Synthesis and Aggressiveness of NB Cells via PARP1‐Mediated E2F1 Transactivation

**Figure 6:**
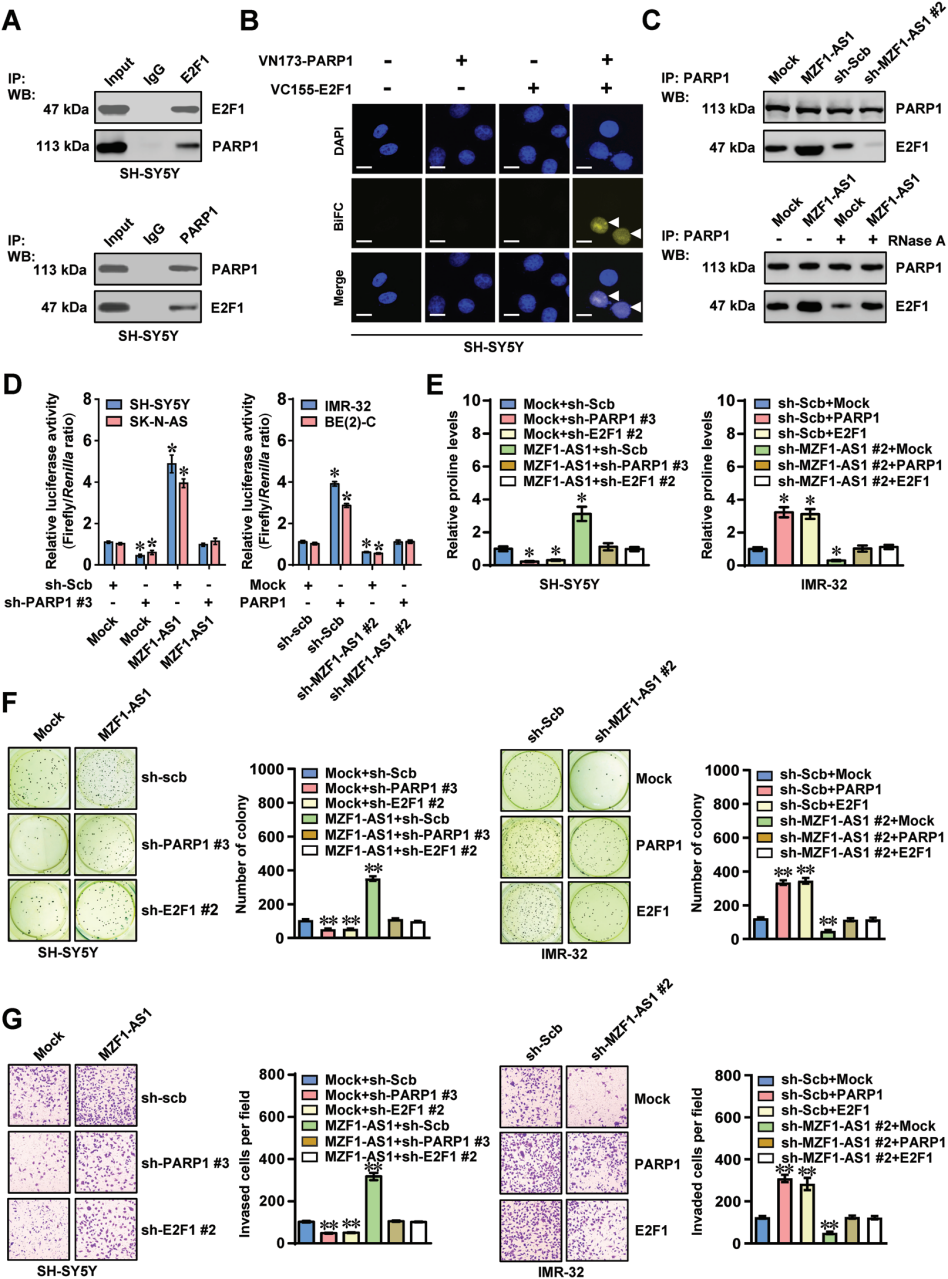
*MZF1‐AS1* facilitates proline synthesis and aggressiveness of NB cells via PARP1‐mediated transactivation of E2F1. A) Co‐IP and Western blot assays showing endogenous interaction between PARP1 and E2F1 in SH‐SY5Y cells. The IgG‐bound protein was taken as negative control. B) Confocal images of BiFC assay indicating the direct interaction between PARP1 and E2F1 (arrowheads) within SH‐SY5Y cells co‐transfected with pBiFC‐PARP1‐VN173 and pBiFC‐E2F1‐VC155. Scale bars: 10 µm. C) Co‐IP and Western blot assays revealing the interaction between PARP1 with E2F1 in SH‐SY5Y cells stably transfected with empty vector (mock), *MZF1‐AS1*, scramble shRNA (sh‐Scb), or sh‐MZF1‐AS1 #2, and those treated with RNase A (20 µg). D) Dual‐luciferase assay indicating the activity of E2F1 in NB cells stably transfected with mock, *MZF1‐AS1*, sh‐Scb, or sh‐MZF1‐AS1#2, and those co‐transfected with sh‐PARP1 #3 or *PARP1* (*n* = 5). E) Proline levels in SH‐SY5Y and IMR‐32 cells stably transfected with mock, *MZF1‐AS1*, sh‐Scb, or sh‐MZF1‐AS1 #2, and those co‐transfected with sh‐PARP1 #3, sh‐E2F1 #2, *PARP1*, or *E2F1* (*n* = 5). F,G) Representative images (left panel) and quantification (right panel) of F) soft agar and G) matrigel invasion assays showing the anchorage‐independent growth and invasion of SH‐SY5Y and IMR‐32 cells stably transfected with mock, *MZF1‐AS1*, sh‐Scb, or sh‐MZF1‐AS1 #2, and those co‐transfected with sh‐PARP1 #3, sh‐E2F1 #2, *PARP1*, or *E2F1* (*n* = 4). Student's *t*‐test compared the difference in panels (D)–(G). *P < 0.05, ** *P* < 0.01 versus mock+sh‐Scb. Data are shown as mean ± s.e.m. (error bars) and representative of three independent experiments in panels (A)–(G).

Since *MZF1‐AS1* bound to PARP1 and regulated E2F1 target genes, we speculated that *MZF1‐AS1* might act as a lncRNA linking PARP1 and E2F1. Endogenous physical interaction between PARP1 and E2F1 was observed in SH‐SY5Y cells (**Figure** **6**A). Bimolecular fluorescence complementation (BiFC) assay, one method observing direct molecular partners,^19^ revealed distinct fluorescence of PARP1 and E2F1 within nucleus of SH‐SY5Y cells (Figure 6B). Notably, knockdown or ectopic expression of *E2F1* abolished the alteration in E2F1 enrichment and target gene expression induced by overexpression or silencing of *PARP1* in SH‐SY5Y and IMR‐32 cells, respectively (Figure S6A–F, Supporting Information). The BRCT‐WGR (376‐662 aa) of PARP1 and DNA binding domain (DBD, 127–192 aa) of E2F1 protein were crucial for their interaction (Figure S7A,B, Supporting Information). Endogenous poly(ADP‐ribosyl)ation (PARylation) of E2F1 was observed in SH‐SY5Y cells, which was enhanced and reduced by treatment with activator (H_2_O_2_) or small molecule inhibitor (PJ‐34) of PARP,^20^ respectively (Figure S7C, Supporting Information). However, the PARylated E2F1 levels were not altered by overexpression or silencing of *PARP1* and *MZF1‐AS1* in SH‐SY5Y cells (Figure S7C,D, Supporting Information). Forced overexpression or knockdown of *MZF1‐AS1* facilitated and suppressed the interaction between PARP1 and E2F1, respectively (Figure 6C), and treatment with RNase A abolished the increased PARP1‐E2F1 interaction induced by *MZF1‐AS1* (Figure 6C). Meanwhile, no direct interaction was observed between GST‐tagged E2F1 protein and *MZF1‐AS1* (Figure S7E, Supporting Information). In addition, knockdown or ectopic expression of *PARP1*, but not pretreatment with PJ‐34 or H_2_O_2_, abolished the increased and decreased E2F1 transactivation induced by overexpression or silencing of *MZF1‐AS1*, respectively (Figure 6D; Figure S7F, Supporting Information). Moreover, knockdown or overexpression of *PARP1* and *E2F1* abolished the increased and decreased ^13^C glutamine‐to‐proline conversion, proline levels, protein synthesis, anchorage‐independent growth, and invasion of NB cells induced by stable ectopic expression or silencing of *MZF1‐AS1* (Figure 6E–G; Figure S8A, Supporting Information). These data indicated that *MZF1‐AS1* facilitated proline synthesis and aggressiveness of NB cells via PARP1‐mediated E2F1 transactivation.

### 2.6 Therapeutic Blocking *MZF1‐AS1*‐PARP1 Interaction Inhibits Proline Synthesis and NB Progression

**Figure 7:**
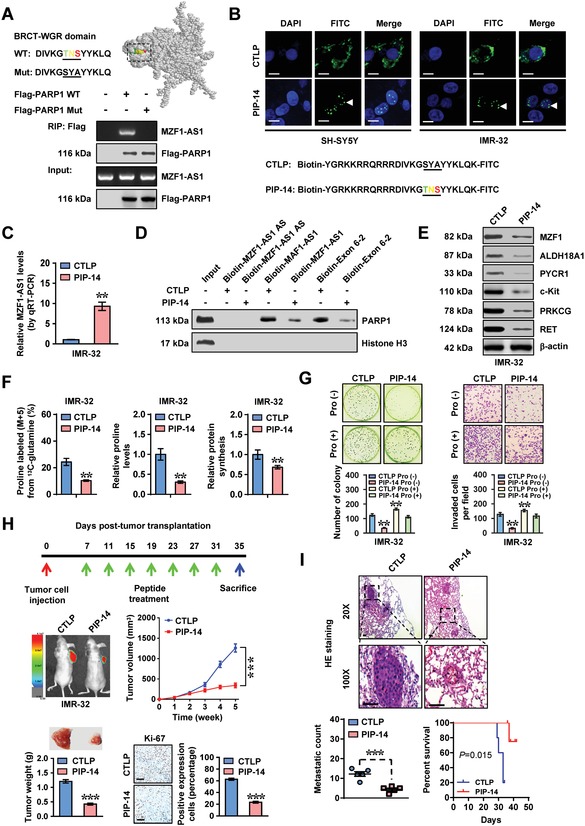
Therapeutic blocking *MZF1‐AS1*‐PARP1 interaction inhibits proline synthesis and NB progression. A) Solution structure (upper panel) for BRCT‐WGR domain of PARP1 analyzed by Jmol program (http://www.jmol.org). RIP assay indicating the interaction of Flag‐tagged wild‐type (WT) or mutant (Mut) PARP1 with *MZF1‐AS1* in IMR‐32 cells. B) Representative images showing the distribution of FITC‐labeled mutant control peptide (CTLP) or inhibitory peptide (PIP‐14, 40 µmol L^−1^) in IMR‐32 cells (at 48 h), with nuclei staining by DAPI. Scale bars: 10 µm. C) Real‐time qRT‐PCR (normalized to input) indicating the levels of *MZF1‐AS1* pulled down by biotin‐labeled CTLP or PIP‐14 (40 µmol L^−1^) from IMR‐32 cells (*n* = 4). D) Biotin‐labeled RNA pull‐down assay revealing the interaction of sense or antisense (AS) *MZF1‐AS1* with PARP1 in IMR‐32 cells treated with CTLP or PIP‐14 (40 µmol L^−1^). E) Western blot showing the expression of *MZF1‐AS1* target genes in IMR‐32 cells treated with CTLP or PIP‐14 (20 µmol L^−1^) for 48 h. F) ^13^C glutamine‐to‐proline conversion (left panel), proline levels (middle panel), and global protein synthesis (right panel) in IMR‐32 cells treated with CTLP or PIP‐14 (20 µmol L^−1^) for 48 h (*n* = 5). G) Representative images and quantification of soft agar (left panel) and matrigel invasion (right panel) assays indicating the anchorage‐independent growth and invasion of IMR‐32 cells treated with CTLP or PIP‐14 (20 µmol L^−1^) for 48 h (*n* = 5), with or without proline (Pro, 3 × 10^−3^ m) supplementation. H) Representative images (left middle panel), in vivo growth curve (right middle panel), tumor weight (left lower panel), and Ki‐67 immunostaining (right lower panel) of xenograft tumors formed by subcutaneous injection of IMR‐32 into the dorsal flanks of nude mice (*n* = 5 per group) that treated with intratumoral administration of CTLP or PIP‐14 (3 mg kg^−1^) as indicated (upper panel). I) H&E staining images and metastatic counts of lungs (upper and left lower panels) and Kaplan–Meier curves (right lower panel) of nude mice (*n* = 5 per group) treated with tail vein injection of IMR‐32 cells and CTLP or PIP‐14 (3 mg kg^−1^). Student's *t*‐test and analysis of variance compared the difference in panel (C) and panels (F)–(I). Log‐rank test for survival comparison in panel (I). ** *P* < 0.01, *** *P* < 0.001 versus CTLP or CTLP Pro (–). Data are shown as mean ± s.e.m. (error bars) and representative of three independent experiments in panels (A)–(G).

Bioinformatic analysis using catRAPID^21^ and RNABindRPuls^22^ programs indicated potential involvement of 80 conserved residues, especially three amino acid residues (TNS), within BRCT‐WGR domain of PARP1 in binding *MZF1‐AS1* (Figure S8B,C and Table S2, Supporting Information). Mutation of these residues abolished the binding of PARP1 to *MZF1‐AS1* in IMR‐32 cells (**Figure** **7**A). Administration of cell‐penetrating fluorescein isothiocyanate (FITC)‐labeled PARP1 inhibiting peptide with 14 amino acids in length (PIP‐14) resulted in its obvious nuclear enrichment in IMR‐32 cells (Figure 7B). Biotin‐labeled peptide pull‐down assay revealed the binding of PIP‐14 to *MZF1‐AS1* (Figure 7C). In addition, PIP‐14 treatment attenuated the interaction of PARP1 with *MZF1‐AS1* (Figure 7D), and led to decrease in downstream gene expression, ^13^C glutamine‐to‐proline conversion, proline levels, protein synthesis, viability, anchorage‐independent growth, and invasion of IMR‐32 cells (Figure 7E–G; Figure S8D, Supporting Information). Meanwhile, supplementation of exogenous proline partially prevented the IMR‐32 cells from decrease of viability, growth, and invasion induced by PIP‐14 (Figure 7G; Figure S8D, Supporting Information). Intratumoral administration of PIP‐14, but not of mutant control peptide, decreased the growth, weight, fluorescence signals, and Ki‐67 proliferation index of IMR‐32 cells formed in subcutaneous xenograft tumors in nude mice (Figure 7H). Treatment with PIP‐14 led to less lung metastatic counts and longer survival time of nude mice with tail vein injection of IMR‐32 cells (Figure 7I; Figure S8E, Supporting Information). These results demonstrated that blocking *MZF1‐AS1*‐PARP1 interaction suppressed the proline synthesis and NB progression.

### 2.7 Therapeutic Knockdown of *MZF1‐AS1* Inhibits Proline Synthesis and NB Progression

To further assess the therapeutic efficacy of lentivirus‐mediated *MZF1‐AS1* knockdown on tumor progression in vivo, nude mice were treated with subcutaneous or tail vein injection of IMR‐32 cells stably expressing red fluorescent protein. Administration of lentivirus‐mediated shRNA against *MZF1‐AS1* (sh‐MZF1‐AS1 #2) dramatically reduced the growth, weight, fluorescence signals, Ki‐67 proliferation index, *MZF1‐AS1*, and downstream gene expression, and proline levels of subcutaneous xenograft tumors in nude mice (Figure S9A–E, Supporting Information). In experimental metastasis assay, nude mice treated with tail vein administration of lentivirus‐mediated sh‐MZF1‐AS1#2 presented lower fluorescence signals, fewer lung metastatic counts, and longer survival time (Figure S9F, Supporting Information). These data indicated that lentivirus‐mediated *MZF1‐AS1* knockdown inhibited proline synthesis and NB progression.

### 2.8 High Expression of *PARP1*, *E2F1*, or Target Gene is Associated with Poor Outcome of NB

**Figure 8:**
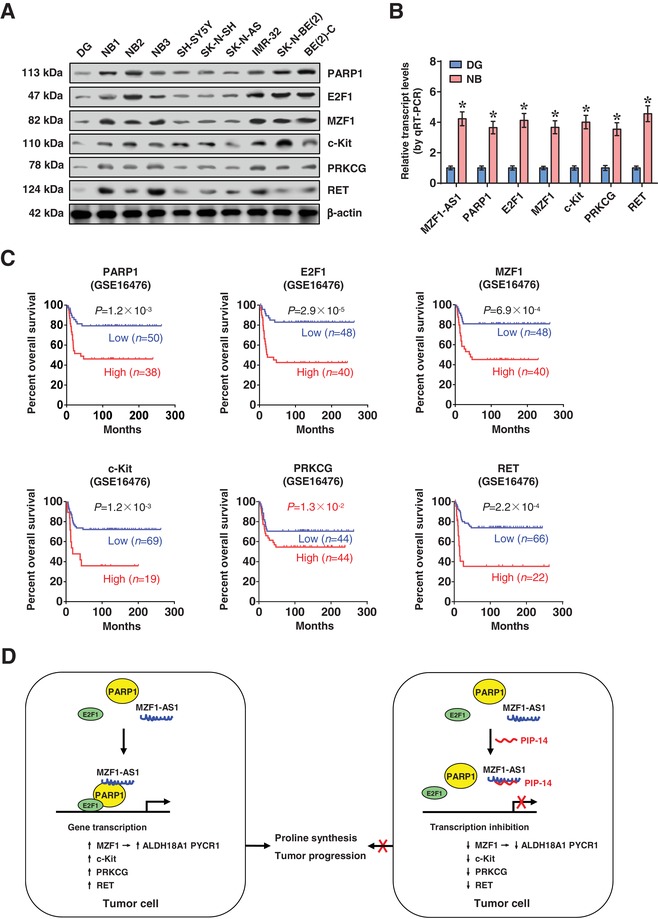
High expression of *PARP1*, *E2F1*, and target genes is associated with poor outcome of NB. A) Western blot and B) real‐time qRT‐PCR (normalized to β‐actin) assays indicating the expression of *MZF1‐AS1*, *PARP1*, *E2F1*, and target genes in normal dorsal root ganglia (DG, *n* = 10), NB tissues (*n* = 42), and NB cell lines. C) Kaplan–Meier curves showing overall survival of 88 (GSE16476) NB cases with low or high expression levels of *PARP1* (cutoff value = 380.6), *E2F1* (cutoff value = 108.1), *MZF1* (cutoff value = 226.6), *c‐Kit* (cutoff value = 145.3), *PRKCG* (cutoff value = 13.6), or *RET* (cutoff value = 94.3). D) The mechanisms underlying *MZF1‐AS1*‐promoted proline synthesis and tumor progression: MZF1 promotes expression of proline synthetic genes, while lncRNA *MZF1‐AS1* directly binds PARP1 to facilitate its interaction with E2F1, resulting in transactivation of E2F1, upregulation of *MZF1* and other oncogenic genes, and promotion of proline synthesis and tumor progression. Student's *t*‐test compared difference in panel (B). Log‐rank test for survival comparison in panel (C). *P < 0.05 versus DG. Data are shown as mean ± s.e.m. (error bars) and representative of three independent experiments in panels (A) and (B).

Higher expression of *MZF1‐AS1*, *PARP1*, *E2F1*, *MZF1*, *c‐Kit*, *PRKCG*, and *RET* was observed in NB tissues, than that in normal dorsal root ganglia (**Figure** **8**A,B). Kaplan–Meier survival analysis indicated that high expression of *PARP1* (*P* = 1.2 × 10^−3^ and *P* = 5.6 × 10^−15^), *E2F1* (*P* = 2.9 × 10^−5^ and *P* = 3.9 × 10^−17^), *MZF1* (*P* = 6.9 × 10^−4^ and *P* = 5.8 × 10^−3^), *c‐Kit* (*P* = 1.2 × 10^−3^ and *P* = 1.3 × 10^−4^), *PRKCG* (*P* = 1.3 × 10^−2^ and *P* = 3.3 × 10^−3^), or *RET* (*P* = 2.2 × 10^−4^ and *P* = 1.7 × 10^−6^) was associated with poor outcome of NB patients (GSE16476 and GSE62564, Figure 8C; Figure S10, Supporting Information). These results indicated that high expression of *PARP1, E2F1*, or target genes was associated with poor outcome of NB.

## 3 Discussion

As nutrients, amino acids are required for protein synthesis, growth, and invasiveness of tumor cells.^23^ However, integrative screening of transcriptional regulators of amino acid metabolic genes in NB remains unknown. In this study, we identify MZF1 as a transcription factor essential for expression of proline synthetic genes *ALDH18A* and *PYCR1*, without impact on proline catabolism gene expression. In human cancers, *ALDH18A1* and *PYCR1* are frequently overexpressed and associated with poor survival of patients,^3^ while depletion of *ALDH18A1* or *PYCR1* diminishes the proline levels and tumorigenesis.^15^, ^24^ In addition, we discover *MZF1‐AS1* as an oncogenic lncRNA associated with poor outcome of NB and other types of tumors. *MZF1‐AS1* facilitates the interaction of PARP1 with E2F1, resulting in upregulation of downstream target genes associated with tumor progression (Figure 8D), such as *MZF1*,^25^ *c‐Kit*,^26^ *PRKCG*,^27^ and *RET*.^28^ As downstream genes of MZF1, *ALDH18A1*, and *PYCR1* exert important functions in *MZF1‐AS1*‐mediated proline synthesis, while proline itself may play a determinative role in the aggressiveness of NB cells.

PARP1, the most abundant enzyme of PARP family, is able to transfer ADP‐ribose units from nicotinamide adenine dinucleotide (NAD^+^) to nuclear proteins,^29^ and regulates gene expression,^30^ chromatin remodeling,^31^ and genomic stability.^32^ Through PARylation of transcription factors such as Yin Yang 1^33^ and activating protein 2,^34^ PARP1 is involved in regulation of gene transcription. PARP1 is associated with tumorigenesis of lung cancer^35^ and breast cancer,^36^ and has been established as a validated target for cancer therapy.^37^ Recent studies show that PARP1 serves as a RNA‐binding protein. For example, *Lnc_bc060912* interacts with PARP1 and nucleophosmin 1 in lung carcinoma cells,^38^ while spliced‐transcript endothelial‐enriched lncRNA (*STEEL*) facilitates the expression of Kruppel like factor 2 via recruiting PARP1 to its promoter.^39^ In this study, we found that PARP1 bound to *MZF1‐AS1*, and tumor promoting functions of *MZF1‐AS1* were mediated, at least in part, through interacting with PARP1 in NB cells. Mechanistically, *MZF1‐AS1* interacted with BRCT‐WGR domain of PARP1 to facilitate its interaction with E2F1, resulting in transactivation of E2F1. A cell‐penetrating small peptide antagonizing *MZF1‐AS1*‐PARP1 interaction was potent in suppressing proline synthesis, tumorigenesis, and aggressiveness, suggesting a potential therapeutic approach for NB.

As a member of E2F transcription factor family, E2F1 regulates gene expression via recognizing an 8 nt motif (TTTSSCGC) within promoters.^40^ High *E2F1* levels are positively associated with advanced stages and poor prognosis of cancers.^41^ Apart from its roles in cell cycle progression, E2F1 contributes to epithelial–mesenchymal transition, metastasis, and invasion of tumors.^42^, ^43^, ^44^ In melanoma, E2F1 enhances metastasis of tumor cells by upregulating vascular endothelial growth factor C.^43^ In addition, E2F1 promotes the invasion and metastasis of small cell lung cancer via regulating matrix metallopeptidase 9 (*MMP‐9*) and *MMP‐16*.^44^ Meanwhile, silencing of *E2F1* attenuates tumor invasion and pulmonary metastasis in melanoma.^45^ In this study, we found that E2F1 facilitated the expression of genes involved in tumor progression, including typical target genes (e.g., *MZF1*, *ALDH18A*, and *PYCR1*) associated with cell cycle, and those not affected by G1/S transition via serum stimulation, which was in line with reported action modes of E2F1.^46^ The activity of E2F1 is regulated by its protein partners. For example, DNA topoisomerase II binding protein 1 inhibits the localization and activity of E2F1.^47^ In this study, our findings indicated that PARP1 served as a co‐factor of E2F1 in regulating target gene expression. Previous studies reveal that E2F1 can be PARylated by PARP1,^20^ and further mapping of PARP1 protein reveals the necessity of BRCT‐WGR domain for its interaction with E2F1.^48^ However, conflicting studies report that PARP1 is not able to mediate the PARylation of E2F1 in vitro, mainly due to varied detection conditions.^48^ Our evidence showed that DBD of E2F1 mediated its interaction with PARP1, and PARP1 facilitated the transactivation of E2F1 in a PARylation‐independent manner. Importantly, we demonstrated that *MZF1‐AS1* increased the transactivation of E2F1, suggesting the crucial roles of E2F1 in *MZF1‐AS1*‐mediated downstream gene expression.

In summary, we have identified that transcription factor MZF1 and its derived lncRNA *MZF1‐AS1* are associated with poor outcome of NB patients, and exert oncogenic roles in regulating proline synthesis and tumor progression. Mechanistically, MZF1 regulates the expression of proline synthetic genes, while *MZF1‐AS1* interacts with PARP1 to enhance its interaction with E2F1, resulting in upregulation of *MZF1* and other oncogenic genes associated with tumor progression. Blocking *MZF1‐AS1*‐PARP1 interaction or *MZF1‐AS1* knockdown exhibits a promising prospect in NB treatment. This study extends our knowledge about the regulation of proline synthesis and tumor progression by transcription factor and its derived lncRNA, and suggests that *MZF1‐AS1*/PARP1/E2F1 axis may be a therapeutic target for tumors. Further investigation is warranted to explore the roles of this axis in proline synthesis in other types of tumors, and elucidate the functions and regulatory mechanisms of aspartic acid and glutamic acid in NB progression.

## 4 Experimental Section

*Cell Lines, Animal Experiments, and Human Tissue Samples*: Cell lines were cultured as recommended by American Type Culture Collection (Rockville, MD) and Children's Oncology Group Cell Bank (Lubbock, TX), and authenticated by short tandem repeat profiling. Animal experiments were carried out in accordance with NIH Guidelines for the Care and Use of Laboratory Animals, and approved by the Animal Care Committee of Tongji Medical College (approval number: Y20080290). The Institutional Review Board of Tongji Medical College approved human tissue study (approval number: 2011‐S085). All procedures were carried out in accordance with approved guidelines. Written informed consent was obtained from all legal guardians of patients.

*Co‐Immunoprecipitation (Co‐IP)*: Co‐IP assay was performed as previously reported,^8^, ^49^ with antibodies specific for PARP1 (ab227244), E2F1 (ab179445), Flag (ab125243), HA (ab9110, Abcam Inc., Cambridge, MA), or PAR (ALX‐804‐220‐R100, Alexis, San Diego, CA). The bead‐bound proteins were released and analyzed by Western blot.

*Cellular Growth, Cell Cycle, and Invasion Assays*: The 2‐(4,5‐dimethyltriazol‐2‐yl)‐2,5‐diphenyl tetrazolium bromide (MTT; Sigma, St. Louis, MO) colorimetric,^7^, ^8^, ^49^ flow cytometric,^7^ colony formation,^50^ soft agar,^7^, ^8^, ^49^ and matrigel invasion^7^, ^8^, ^49^ assays were undertaken to measure in vitro viability, cell cycle progression, growth, and invasion of tumor cells.

*Immuno‐Histochemistry*: Immuno‐histochemical staining and quantitative evaluation were performed as previously reported,^8^, ^49^ with antibodies specific for Ki‐67 (sc‐22 835, Santa Cruz Biotechnology, Santa Cruz, CA, 1:200 dilution).

*Statistical Analysis*: All data were shown as mean ± standard error of the mean (s.e.m.). Cutoff values were determined by average gene expression levels. Student's *t*‐test, analysis of variance, and χ^2^ analysis were applied to compare difference. Fisher's exact test was applied to analyze statistical significance of overlap between two gene lists. Pearson's correlation coefficient was applied for analyzing relationship among gene expression. Log‐rank test was used to assess survival difference. All statistical tests were two‐sided and considered statistically significant, when false discovery rate (FDR)‐corrected *P* values were less than 0.05.

Detailed Experimental Section is described in the Supporting Information.

## Conflict of Interest

The authors declare no conflict of interest.

## Supporting information

Supplementary
