# Supplementary material for: Therapeutic Targeting of MZF1‐AS1/PARP1/E2F1 Axis Inhibits Proline Synthesis and Neuroblastoma Progression
Source: Adv Sci (Weinh). 2019 Aug 10;6(19):1900581. doi: 10.1002/advs.201900581 (PMC6774027; doi:10.1002/advs.201900581)
Supplement: Supplementary file 1 — Supplementary [file ADVS-6-1900581-s001.pdf]

Copyright WILEY-VCH Verlag GmbH & Co. KGaA, 69469 Weinheim, Germany, 2019.

Supporting Information

for *Adv. Sci.*, DOI: 10.1002/advs.201900581

Therapeutic Targeting of *MZF1-AS1*/PARP1/E2F1 Axis Inhibits Proline Synthesis and Neuroblastoma Progression

Erhu Fang, Xiaojing Wang, Feng Yang, Anpei Hu, Jianqun Wang, Dan Li, Huajie Song, Mei Hong, Yanhua Guo, Yang Liu, Hongjun Li, Kai Huang, Liduan Zheng, and Qiangsong Tong**

Copyright WILEY-VCH Verlag GmbH & Co. KGaA, 69469 Weinheim, Germany, 2019.

Supporting Information

Therapeutic targeting of *MZF1-AS1*/PARP1/E2F1 axis inhibits proline synthesis and neuroblastoma progression

*Erhu Fang, Xiaojing Wang, Feng Yang, Anpei Hu, Jianqun Wang, Dan Li, Huajie Song, Mei Hong, Yanhua Guo, Yang Liu, Hongjun Li, Kai Huang, Liduan Zheng *, Qiangsong Tong **

Detailed Experimental Section

Cell culture: Human NB cell lines SH-SY5Y (CRL-2266), SK-N-SH (HTB-11), SK-N-AS (CRL-2137), IMR-32 (CCL-127), SK-N-BE(2) (CRL-2271), NB-1643, and BE(2)-C (CRL-2268), colon cancer SW480 (CCL-228) cells, cervical cancer SiHa (HTB-35) cells, and embryonic kidney HEK293T cells (CRL-1573) were obtained from American Type Culture Collection (Rockville, MD) and Children's Oncology Group Cell Bank (Lubbock, TX). Cell lines were authenticated by short tandem repeat profiling, and used within 6 months after resuscitation of frozen aliquots. Mycoplasma contamination was regularly examined using Lookout Mycoplasma PCR Detection Kit (Sigma, St. Louis, MO). Cells were cultured in Dulbecco's modified Eagle's medium (DMEM) containing 10% fetal bovine serum (FBS, Gibco, Grand Island, NY) and 2 mM L-glutamine, and supplemented with L-aspartic acid, L-glutamic acid, or proline (Sigma). Cells were starved with 0.1% FBS, stimulated with 10% FBS containing media, and treated with H_2O_2 or PJ-34 (Sigma) as indicated.

RNA isolation and real-time qRT-PCR: Nuclear and cytoplasmic RNA was extracted according to the instruction of RNA Subcellular Isolation Kit (Active Motif, Carlsbad, CA). Total RNA was isolated with RNAiso Plus (Takara Bio Inc., Japan). Reverse transcription reactions were undertaken with PrimeScript™ RT reagent Kit (Takara Bio Inc.). Real-time PCR was performed with SYBR® Premix Ex Taq™ II (Takara Bio Inc.) and primer sets (Table S3, Supporting Information). The transcript levels were analyzed by $2^{-\Delta\Delta Ct}$ method.

Northern blot: To prepare the *MZF1-AS1* probe, the 185-bp PCR product (Table S3, Supporting Information) was *in vitro* transcribed using DIG Labeling Kit (MyLab Corporation, Beijing, China) and T7 RNA polymerase, and treated with RNase-free DNase I. For Northern blot, 20 μg of total RNA was separated on 3-(N-morpholino)propanesulfonic acid (MOPS)-buffered 2% (w/v) agarose gel containing 1.2% (v/v) formaldehyde under denaturing conditions for 4 hrs at 80 V, and transferred to Hybond-N+ membrane (Pall Corp., Port Washington, NY). Prehybridization was carried out at 65°C for 30 min in DIG Easy Hyb solution (Roche, Indianapolis, IN). Hybridization was performed at 65°C for 16-18 hrs. Blots were washed stringently, detected by anti-digoxigenin (DIG) antibody, and recorded on X-ray films with chemiluminescence substrate CSPD (Roche).

RNA-FISH: Biotin-labeled probes for *MZF1-AS1*, glyceraldehyde 3-phosphate dehydrogenase (*GAPDH*), and *U1* were generated by *in vitro* transcription of PCR products (Table S3, Supporting Information) using DIG Labeling Kit (MyLab Corporation). Cells were seeded on coverslips, fixed in 4% paraformaldehyde for 15 min, and incubated with 40 nmol/L FISH probe in hybridization buffer (100 mg/ml dextran sulfate, 10% formamide in 2×SSC) at 80°C for 2 min. Hybridization was performed at 55°C for 2 hrs, with or without RNase A (20 μg) treatment. The signals of *MZF1-AS1* were detected by Fluorescent In Situ Hybridization Kit (RiboBio, Guangzhou, China), with nuclei counterstained with 4',6-diamidino-2-phenylindole (DAPI).

Fluorescence immunocytochemical staining: Tumor cells were grown on coverslips, incubated with 5% milk for 1 hr, and treated with antibodies specific for PARP1 (ab227244, Abcam Inc., Cambridge, MA; 1:100 dilution) at 4°C overnight. Then, coverslips were treated with Alexa Fluor 594 goat anti-rabbit IgG (1:1000 dilution) and DAPI (300 nmol/L) staining. The images were photographed under a Nikon A1Si Laser Scanning Confocal Microscope (Nikon Instruments Inc, Japan).

Over-expression or knockdown of genes: Human *MZF1-AS1* cDNA (2606 bp), *PARP1* cDNA (3045 bp), *E2F1* cDNA (1314 bp), and their truncations were amplified from NB tissues by PCR primers (Table S3, Supporting Information) and inserted into pcDNA3.1 (Invitrogen, Carlsbad, CA), pCMV-3Tag-1C (Addgene, Cambridge, MA), and pCMV-HA (Addgene), respectively. Mutation of *PARP1* was prepared with GeneTailor™ Site-Directed Mutagenesis System (Invitrogen), using PCR primers indicated in Table S4, Supporting Information. Oligonucleotides specific for shRNAs against *MZF1*, *MZF1-AS1*,

ALDH18A1, *PYCR1*, *PARP1*, and *E2F1* (Table S5, Supporting Information) were inserted into lentiviral vector GV298 (Genechem Co., Ltd, Shanghai, China). After selection for neomycin or puromycin (Invitrogen) resistance, stable cell lines were obtained.

Rescue of target gene expression: To restore target gene expression induced by *MZF1-AS1* knockdown, tumor cells were transfected with *PARP1* or *E2F1* expression vector. To rescue gene expression altered by over-expression of *MZF1-AS1*, shRNAs specific for *ALDH18A1*, *PYCR1*, *PARP1*, or *E2F1* were transfected into tumor cells with Genesilencer Transfection Reagent (Genlantis, San Diego, CA). Empty vector and sh-Scb were applied as controls (Table S5, Supporting Information).

Lentiviral packaging: Lentiviral vectors were co-transfected with packaging plasmids psPAX2 and pMD2G (Addgene) into HEK293T cells. Infectious lentivirus was harvested at 36 and 60 hrs after transfection, and filtered through 0.45 μm PVDF filters. Recombinant lentivirus was concentrated 100-fold by ultracentrifugation (2 hrs at 120,000 g). Lentivirus-containing pellet was dissolved in phosphate buffer saline (PBS) and injected in mice within 48 hrs.

RNA-seq assay: Total RNA of tumor cells (1×10^6) was extracted according to manual of TRIzol® reagent (Life Technologies, Inc., Gaithersburg, MD). Library preparation and transcriptome sequencing on an Illumina HiSeq X Ten platform were carried out at Novogene Bioinformatics Technology Co., Ltd. (Beijing, China) to generate 100-bp paired-end reads. HTSeq v0.6.0 was applied in counting the numbers of read mapping to each gene, and fragments per kilobase of transcript per million fragments mapped (FPKM) of each gene were calculated. Sequencing results have been deposited in GEO database (accession code GSE133009).

RNA pull-down and mass spectrometry: Biotin-labeled RNAs were *in vitro* transcribed using Biotin RNA Labeling Mix (Roche) and T7 RNA polymerase, treated with RNase-free DNase I, and purified with RNeasy Mini Kit (Qiagen Inc., Redwood City, CA). Nuclear extracts were harvested, resuspended in freshly prepared proteolysis buffer, and incubated with biotin-labeled RNA (10 pmol) and streptavidin-agarose beads (Invitrogen) for 1 hr. Precipitated components were separated using SDS-PAGE, followed by Coomassie blue staining or western blot. Differential bands were harvested for mass spectrometry analysis (Wuhan Institute of Biotechnology, Wuhan, China).

Western blot: Tissue or cellular protein was extracted with 1 × cell lysis buffer (Promega, Madison, WI). Western blot was performed with antibodies (1:1000 dilution) specific for MZF1 (ab64866), ALDH18A1 (ab223713), ODC1 (ab193338), PYCR1 (ab94780), SLC7A5 (ab85226), VARS (ab181175), PYCR2 (ab103535), PYCR3 (H00065263-M01, Novus Biologicals, Centennial, CO), PRODH (ab93210), P5CDH (ab185208), phosphorylated mTOR (p-mTOR^{Ser2448}, ab109268), p-70S6K1 $^{Thr389/412}$ (ab60948), p-4EBP1 ^{Thr37} (ab75767), 4EBP1 (ab2606), PARP1 (ab227244), DHX30 (ab85687), DHX36 (ab70269), NAA15 (ab65107), SAFB (ab97520), SND1 (ab65078), SUPT16H (ab204343), histone H3 (ab5103), E2F1 (ab179445), c-Kit (ab32363), PRKCG (ab154690), RET (ab134100), GST (ab19256), Flag (ab125243), HA (ab9110), GAPDH (ab8245), or β-actin (ab8226, Abcam Inc.).

Cross-linking RIP assay: Tumor cells (1×10^8) were ultraviolet light cross-linked at 254 nm (200 J/cm^2) in PBS and collected by scraping. RIP assay was performed according to instruction of Magna RIP™ RNA-Binding Protein Immunoprecipitation Kit (Millipore, Temecula, CA), with antibodies specific for PARP1 (ab227244) or Flag (ab125243, Abcam Inc.). Co-precipitated RNAs were detected by RT-PCR or real-time quantitative qRT-PCR with specific primers (Table S3, Supporting Information). Total RNAs (input) and isotype antibody (IgG) were applied as controls.

RNA EMSA: A series of *PARP1* truncations were amplified from NB tissues (Table S4, Supporting Information), subcloned into pGEX-6P-1 (Addgene), and transformed into *E. coli* to produce GST-tagged PARP1 proteins. Biotin-labeled RNA probes for exons 1–6 of *MZF1-AS1* were *in vitro* transcribed as described above. RNA EMSA using nuclear extracts or recombinant PARP1 protein was performed

according to manuals of LightShift Chemiluminescent RNA EMSA Kit (Thermo Fisher Scientific, Inc., Waltham, MA).

***In vitro* binding assay:** Biotin-labeled *MZF1-AS1* probe was prepared as described above, and incubated with GST-tagged PARP1 protein. PARP1-*MZF1-AS1* complexes were pulled down using GST beads (Sigma). Protein was detected by SDS-PAGE and western blot, while *MZF1-AS1* was measured by RT-PCR with specific primers (Table S3, Supporting Information).

BiFC assay: Human *PARP1* cDNA (3045 bp) and *E2F1* cDNA (1314 bp) were respectively subcloned into BiFC vectors pBiFC-VN173 and pBiFC-VC155 (Addgene), and co-transfected into tumor cells with Lipofectamine 2000 (Invitrogen) for 24 hrs. The fluorescence emission was observed under a confocal microscope, using excitation and emission wavelengths of 488 and 500 nm, respectively.

Dual-luciferase reporter assay: The promoter fragments of *ALDH18A1* (-281/+225) and *PYCR1* (-471/+106) were amplified from genomic DNA by PCR (Table S4, Supporting Information) and subcloned into pGL3-Basic (Promega). Human *E2F1* luciferase reporter was established by annealing two complementary oligonucleotides containing four canonical E2F1 binding sites (Table S4, Supporting Information) and inserting into pGL3-Basic (Promega). Dual-luciferase assay was performed according to manufacturer's instruction (Promega). Luciferase reporters of AP1, HIF1α, and TP53 were obtained from Qiagen Inc. and Stratagene (La Jolla, CA). Luciferase activity was measured with a luminometer (Lumat LB9507, Berthold Tech., Bad Wildbad, Germany).

ChIP and qPCR assay: ChIP assay was performed according to instruction of EZ-CHIP Kit (Upstate Biotechnology, Temacula, CA). Real-time qPCR was undertaken with SYBR Green PCR Master Mix (Applied Biosystems, Foster City, CA) and primers targeting gene promoters (Table S3, Supporting Information).

Design and synthesis of inhibitory peptides: Inhibitory peptides for blocking interaction between *MZF1-AS1* and PARP1 were designed and synthesized (ChinaPeptides Co. Ltd, Shanghai, China). The 11 amino acid long peptide (YGRKKRRQRRR) from Tat protein transduction domain served as a cell-penetrating peptide. Thus, inhibitory peptides were chemically synthesized by linking with biotin-labeled cell-penetrating peptide at N-terminus and conjugating with FITC at C-terminus. The purity of peptides (larger than 95%) was validated by reversed phase-high performance liquid chromatography assay.

Biotin-labeled peptide pull-down assay: Cellular protein was isolated using 1× cell lysis buffer (Promega), and incubated with biotin-labeled peptide at 4°C overnight. Then, incubation of cell lysates with streptavidin-agarose was undertaken at 4°C for 2 hrs. The beads were extensively washed, and lncRNAs pulled down were measured by real-time qRT-PCR.

Measurement of proline levels and protein synthesis: For [U-$^{13}C_5$] glutamine flux experiments, cells were cultured in glutamine-free DMEM (Sigma) containing 10% FBS and 4 mmol/L L[U-$^{13}C_5$]-glutamine (Cambridge Isotope Labs, Tewksbury, MA) for 18 hrs. The metabolites were extracted by incubating with methanol/acetonitrile/dH_2O (4.5:4.5:1) for 15 min, and sonicated for 30 sec at 4ºC. The supernatants were subjected to liquid chromatography mass spectrometry (LC-MS) analysis. Intracellular proline levels and *de novo* protein synthesis were measured using Proline Colorimetric Assay Kit (Elabscience, Houston, TX) or EZClick™ Global Protein Synthesis Assay Kit (BioVision, Milpitas, CA), according to manufacturer's instruction.

***In vivo* tumorigenesis and aggressiveness assays:** All animal experiments were carried out in accordance with NIH Guidelines for the Care and Use of Laboratory Animals, and approved by the Animal Care Committee of Tongji Medical College (approval number: Y20080290). For *in vivo* tumorigenesis studies, tumor cells (1×10^6) were subcutaneously injected into dorsal flanks of blindly randomized four-week-old

female BALB/c nude mice (*n*=5 per group). The growth and weight of xenograft tumors were detected one month later. In experimental metastasis studies, tail vein injection of tumor cells (4×10^6) was performed in blindly randomized four-week-old female BALB/c nude mice (*n*=5 per group). For *in vivo* therapeutic studies, tumor cells (1×10^6 or 0.4×10^6) were injected into dorsal flanks or tail vein of nude mice, respectively. One week after tumor cell inoculation, mice were blindly randomized and treated by intratumoral or tail vein injection of lentivirus-mediated shRNA (1×10^7 plaque-forming units in 100 μl PBS) or synthesized cell-penetrating peptide (ChinaPeptides Co. Ltd) as indicated. Tumor volume and survival time of each mouse were monitored and recorded. The animals were imaged using In-Vivo Xtreme II small animal imaging system (Bruker Corporation, Billerica, MA).

Patient tissue samples: The Institutional Review Board of Tongji Medical College approved the human tissue study (approval number: 2011-S085). All procedures were carried out in accordance with approved guidelines. Written informed consent was obtained from all legal guardians of patients. The NB tissues were obtained during surgery at Union Hospital of Tongji medical College. Patients with a history of preoperative chemotherapy or radiotherapy were excluded. Human normal dorsal root ganglia tissues were collected from therapeutic abortion. All fresh specimens were frozen in liquid nitrogen, validated by pathological diagnosis, and stored at -80°C until use.

Amino acid profiling: For each sample, 30 mg of tissue was homogenized with zirconia beads at 30 Hz for 3 min, ultrasonicated for 10 min, and centrifugated at 4°C and 16000 g for 15 min. The supernatant was applied to analyze concentrations of amino acids, which was performed by an Agilent 1290 ultra-high performance liquid chromatography (UHPLC) system coupled to an Agilent 6470A Triple Quadrupole MS System (Agilent Technologies, Santa Clara, CA). A Waters BEH Amide column (100 mm × 2.1mm, 1.7 μm) was used for amino compound separation with a flow rate at 0.35 ml/min and column temperature at 55°C. The mobile phase consisted of (A) 10 mM ammonium formate in water, 0.2% formic acid, and 0.08 mM phthalic acid, (B) acetonitrile with 2 mM ammonium formate, 0.2% formic acid, and 0.08 mM phthalic acid. The chromatographic separation was conducted by a gradient elution program as follows: 1 min, 85% B; 4 min, 82% B; 7 min, 60% B; 8 min, 50% B and held to 9 min; 9.1 min, 85% B and held to 12 min. Multiple Reaction Monitoring (MRM) was used to monitor amino acid compounds. All amino acid standards were purchased from Alfa-aesar (Tewksbury, MA). The Agilent MassHunter Quantitation Analysis (version B08) was applied to analyze data.

Data availability: RNA-seq data supporting the results of this study have been deposited in GEO database (https://www.ncbi.nlm.nih.gov/geo/), under accession number GSE133009. Public datasets are available from GEO database (GSE16476, GSE62564, GSE1084, and GSE9891) or TCGA database. All remaining data are presented within the article and Supporting Information Files, and available from the corresponding author upon request.

Supplementary data

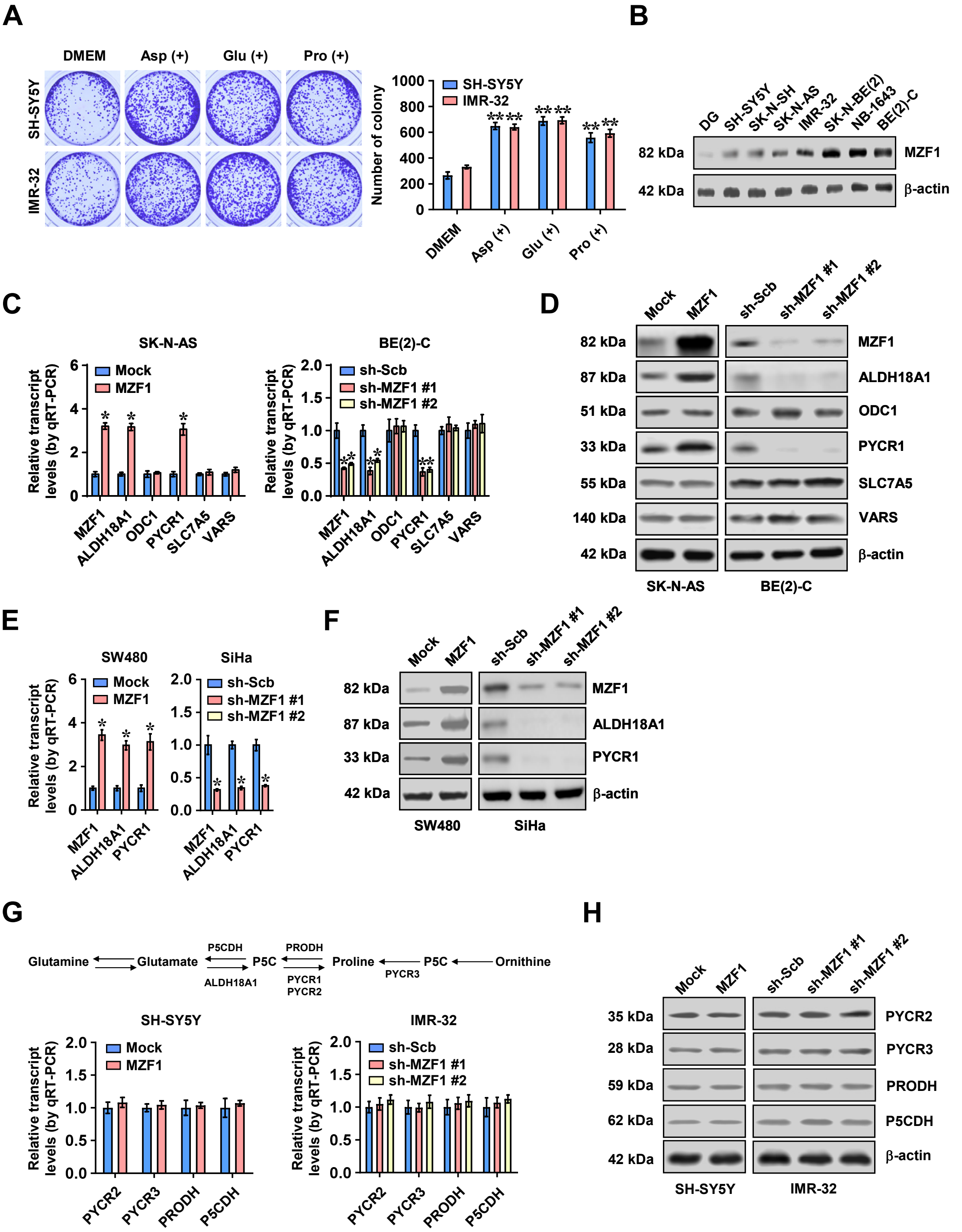

Figure S1. ***MZF1* facilitates the expression of proline synthetic genes in NB. A**) Representative images (left panel) and quantification (right panel) of colony formation assay revealing the growth of SH-SY5Y and IMR-32 cells cultured in DMEM lacking non-essential amino acids, without or with supplementation of L-aspartic acid (Asp, 1 mM), L-glutamic acid (Glu, 1 mM), or proline (Pro, 3 mM). **B**) Western blot assay indicating the MZF1 levels in normal dorsal root ganglia (DG) and NB cell lines. **C** and **D**) Real-time qRT-PCR (C, normalized to β-actin, *n*=4) and western blot (D) assays showing the expression of *MZF1*, *ALDH18A1*, *ODC1*, *PYCR1*, *SLC7A5*, or *VARS* in SK-N-AS and BE(2)-C cells stably transfected with empty vector (mock), *MZF1*, scramble shRNA (sh-Scb), sh-MZF1 #1, or sh-MZF1 #2. **E** and **F**) Real-time qRT-PCR (E, normalized to β-actin, *n*=4) and western blot (F) assays indicating the levels of *MZF1*, *ALDH18A1* and *PYCR1* in colon cancer SW480 cells and cervical cancer SiHa cells transfected with mock, *MZF1*, sh-Scb, sh-MZF1 #1, or sh-MZF1 #2. **G** and **H**) Real-time qRT-PCR (G, normalized to β-actin, *n*=4) and western blot (H) assays showing the levels of proline synthetic and catabolism genes *PYCR2*, *PYCR3*, *PRODH*, or *P5CDH* in SH-SY5Y and IMR-32 cells stably transfected with mock, *MZF1*, sh-Scb, sh-MZF1 #1, or sh-MZF1 #2. Student's *t* test compared difference in **A**, **C**, **E** and **G**. * P<0.05, ** P<0.01 vs. DMEM, mock, or sh-Scb. Data are shown as mean ± s.e.m. (error bars) and representative of three independent experiments in **A**-**H**.

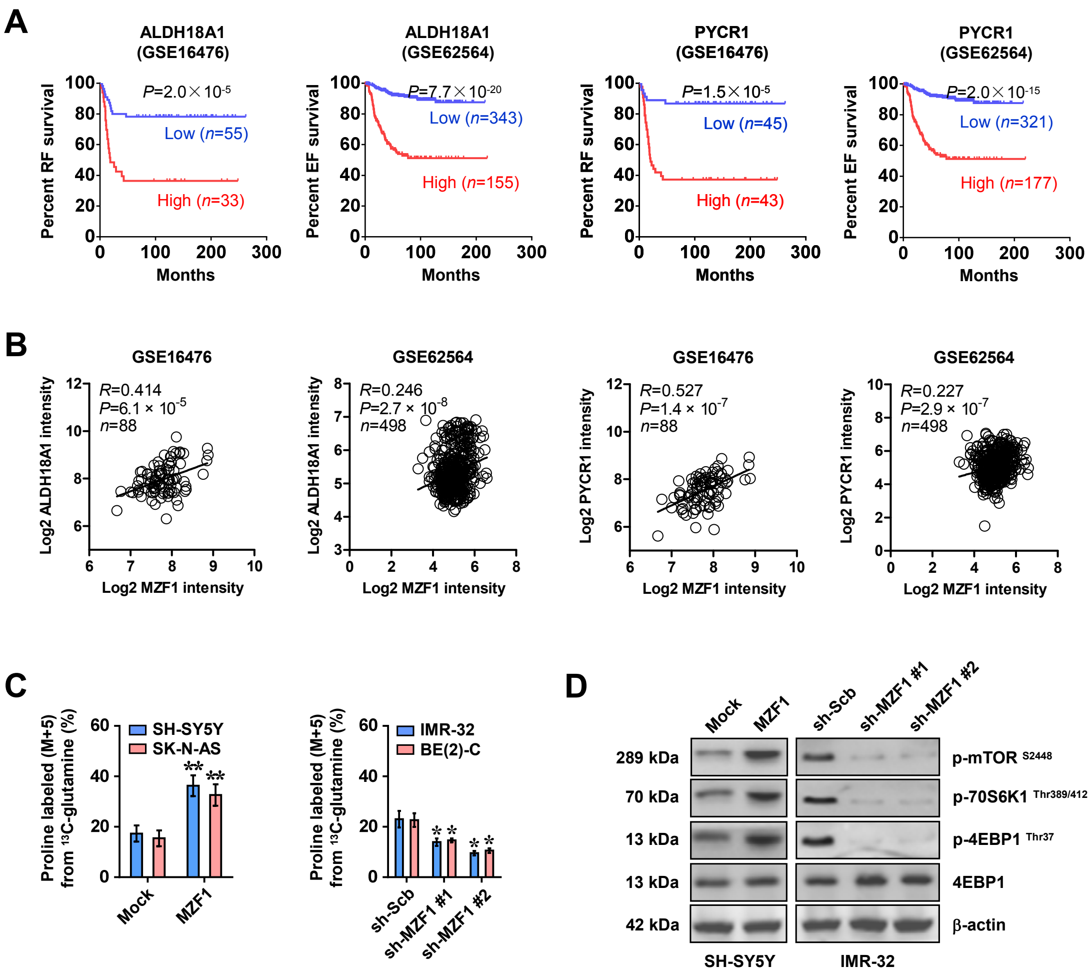

Figure S2. Expression profiles of *MZF1* and target genes in NB tissues and cells. A) Kaplan–Meier survival plots of 88 (GSE16476) and 498 (GSE62564) NB patients with low or high levels of *ALDH18A1* (cutoff values=266.4 and 44.2) or *PYCR1* (cutoff values=199.9 and 50.1). **B**) Positive expression correlation between *MZF1* and *ALDH18A1* or *PYCR1* in 88 (GSE16476) and 498 (GSE62564) NB cases. **C**) LC-MS analysis of ^{13}C glutamine-to-proline flux in SH-SY5Y, SK-N-AS, IMR-32, and BE(2)-C cells stably transfected with empty vector (mock), *MZF1*, scramble shRNA (sh-Scb), sh-MZF1 #1, or sh-MZF1 #2 (n=4). **D**) Western blot indicating the expression of p-mTOR, p-70S6K1, p-4EBP1, or 4EBP1 in SH-SY5Y and IMR-32 cells stably transfected with mock, *MZF1*, sh-Scb, sh-MZF1 #1, or sh-MZF1 #2. Log-rank test for survival comparison in **A**. Pearson's correlation coefficient analysis in **B**. Student's t test compared difference in **C**. * P<0.05, ** P<0.01 vs. mock or sh-Scb. Data are shown as mean ± s.e.m. (error bars) and representative of three independent experiments in **C** and **D**.

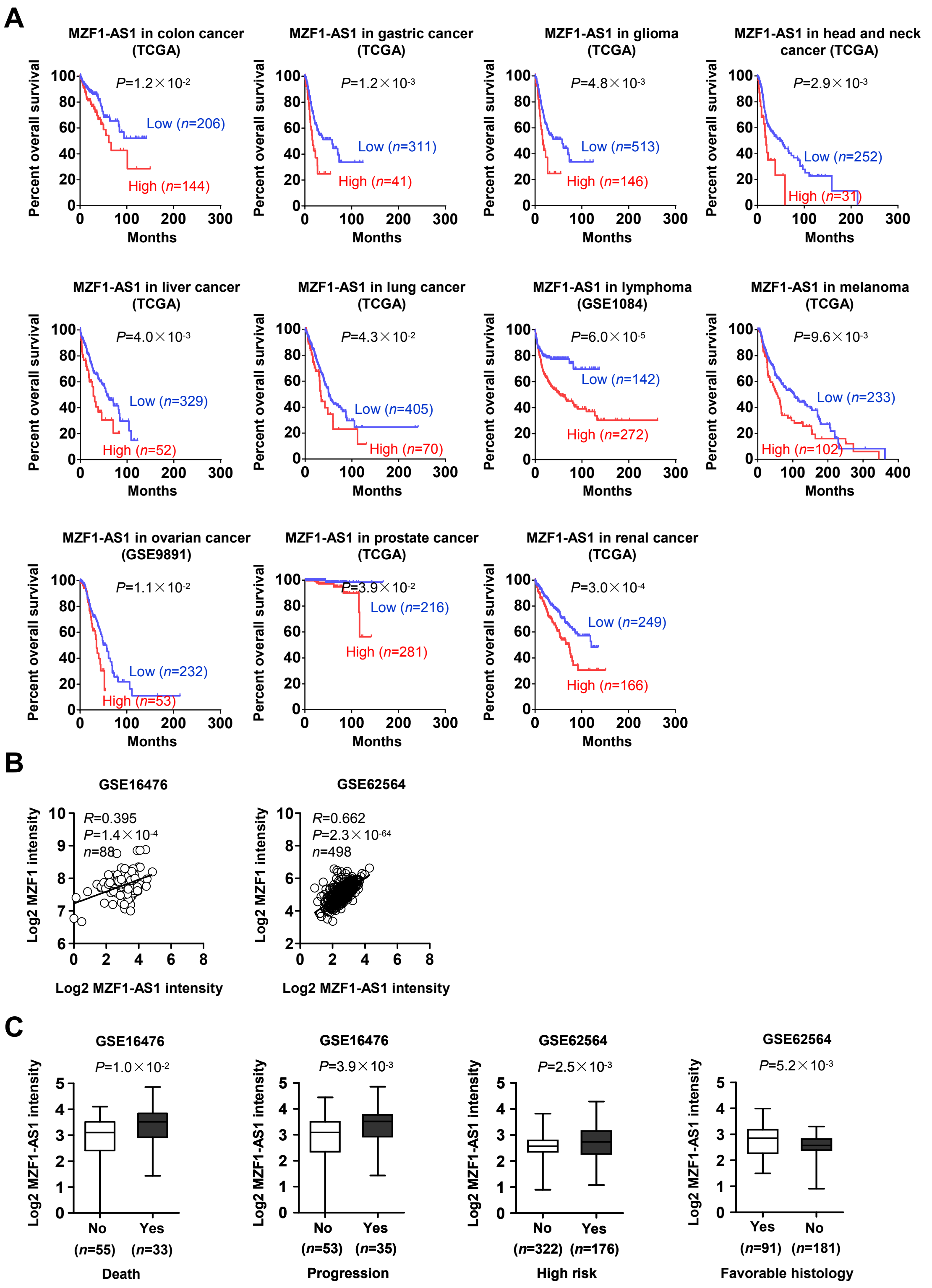

Figure S3. Expression of *MZF1-AS1* in tumor tissues. A) Kaplan–Meier survival plots of patients with low or high levels of *MZF1-AS1* in TCGA or GEO datasets of colon cancer, gastric cancer, glioma, head and neck cancer, liver cancer, lung cancer, lymphoma, melanoma, ovarian cancer, prostate cancer, and renal cancer. **B**) Positive expression correlation between *MZF1-AS1* and *MZF1* in 88 (GSE16476) and 498 (GSE62564) NB cases. **C**) Relative *MZF1-AS1* levels in 88 (GSE16476) and 498 (GSE62564) NB cases with different status of death, clinical progression, high risk, or histology. Log-rank test for survival comparison in **A**. Pearson's correlation coefficient analysis in **B**. Student's *t* test compared difference in **C**. Bars are means and whiskers (min to max) in **C**.

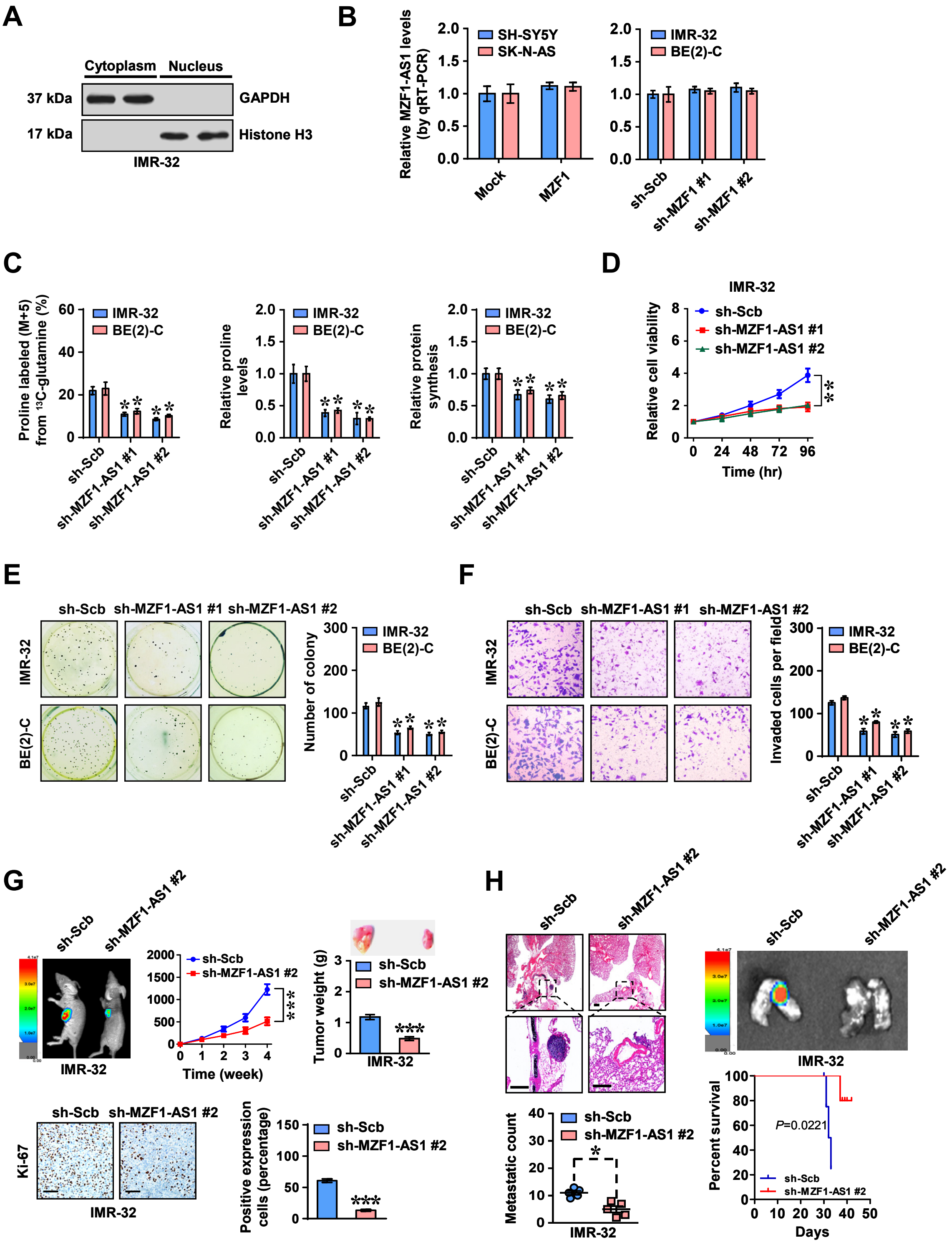

Figure S4. ***MZF1-AS1* facilitates proline synthesis, tumorigenesis and aggressiveness. A**) Western blot assay indicating the expression of GAPDH and histone H3 in cytoplasmic and nuclear fractions of IMR-32 cells. **B**) Real-time qRT-PCR (normalized to β-actin, n=5) assay showing the expression of *MZF-AS1* in NB cells stably transfected with empty vector (mock), *MZF1*, scramble shRNA (sh-Scb), or sh-MZF1. **C**) ^{13}C glutamine-to-proline conversion (left panel), proline levels (middle panel), and *de novo* protein synthesis (right panel) in IMR-32 and BE(2)-C cells stably transfected with sh-Scb or sh-MZF1-AS1 (n=5). **D-F**) MTT colorimetric assay (D), representative images and quantification of soft agar (E) and matrigel invasion (F) assays indicating the viability, anchor-independent growth, and invasion of NB cells stably transfected with sh-Scb or sh-MZF1-AS1 (n=4). **G**) Representative images (left upper panel), *in vivo* growth curve (middle upper panel), weight at the end points (right upper panel), representative images (left lower panel) and quantification (right lower panel) of Ki-67 expression of xenograft tumors formed by subcutaneous injection of IMR-32 cells stably transfected with sh-Scb or sh-MZF1-AS1 #2 into dorsal flanks of nude mice (n=5 per group). **H**) H&E staining (left upper panel), representative images (right upper panel), metastatic counts of lungs (left lower panel), and Kaplan-Meier curves (right lower panel) of nude mice (n=5 per group) treated with tail vein injection of IMR-32 cells stably transfected with sh-Scb or sh-MZF1-AS1 #2. Scale bar: 100 μm. Student's t test compared difference in **B-H**. Log-rank test for survival comparison in **H**. * P<0.05, ** P<0.01, *** P<0.001 vs. sh-Scb. Data are shown as mean ± s.e.m. (error bars) and representative of three independent experiments in **A-F**.

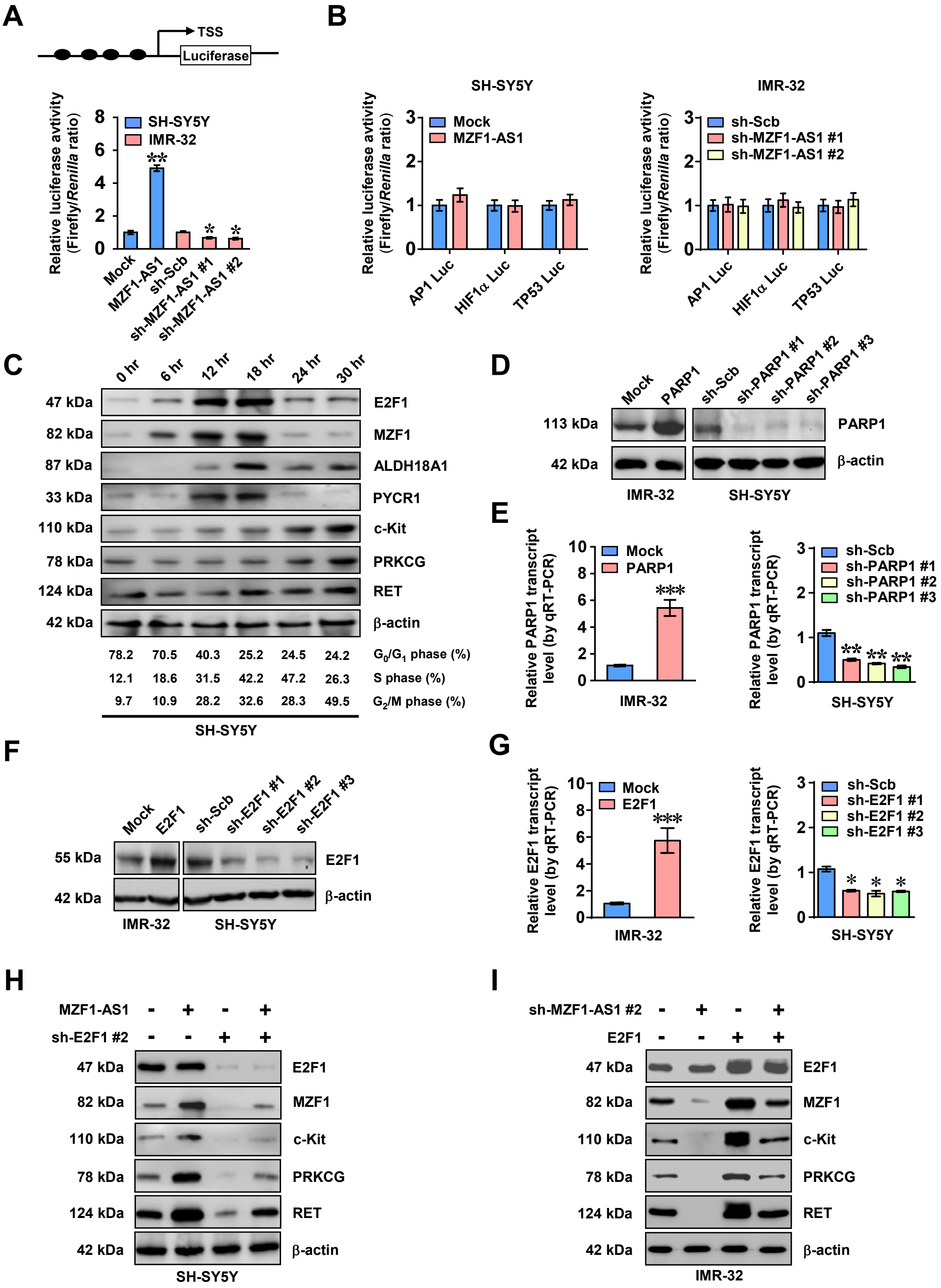

Figure S5. ***MAZ1-AS1* interacts with PARP1 and induces E2F1 transactivation.** **A**) Dual-luciferase assay with a reporter containing four canonical E2F1 binding sites showing the relative activity of E2F1 in SH-SY5Y and IMR-32 cells stably transfected with empty vector (mock), *MZF1-AS1*, scramble shRNA (sh-Scb), or sh-MZF1-AS1 (*n*=4). **B**) Dual-luciferase assay indicating the relative activity of AP1, HIF1α, or TP53 in SH-SY5Y and IMR-32 cells stably transfected with mock, *MZF1-AS1*, sh-Scb, or sh-MZF1-AS1 (*n*=4). **C**) Flow cytometry and western blot assays showing cell cycle phases and expression of E2F1 and target genes in SH-SY5Y cells starved with 0.1% FBS containing media for 30 hrs and then stimulated with 10% FBS containing media followed by collection at indicated time points. **D** and **E**) Western blot (D) and real-time qRT-PCR (E, normalized to β-actin, *n*=4) assays indicating *PARP1* levels in IMR-32 and SH-SY5Y cells transfected with mock, *PARP1*, sh-Scb, or sh-PARP1. **F** and **G**) Western blot (F) and real-time qRT-PCR (G, normalized to β-actin, *n*=4) assays showing the expression of *E2F1* in IMR-32 and SH-SY5Y cells transfected with mock, *E2F1*, sh-Scb, or sh-E2F1. **H** and **I**) Western blot assay indicating protein levels of target genes in SH-SY5Y and IMR-32 cells stably transfected with mock, *MZF1-AS1*, sh-Scb, or sh-MZF1-AS1 #2, and those co-transfected with sh-E2F1 #2 or *E2F1*. Student's *t* test compared difference in **A**, **B**, **E** and **G**. * *P*<0.05, ** *P*<0.01, *** *P*<0.001 vs. mock or sh-Scb.

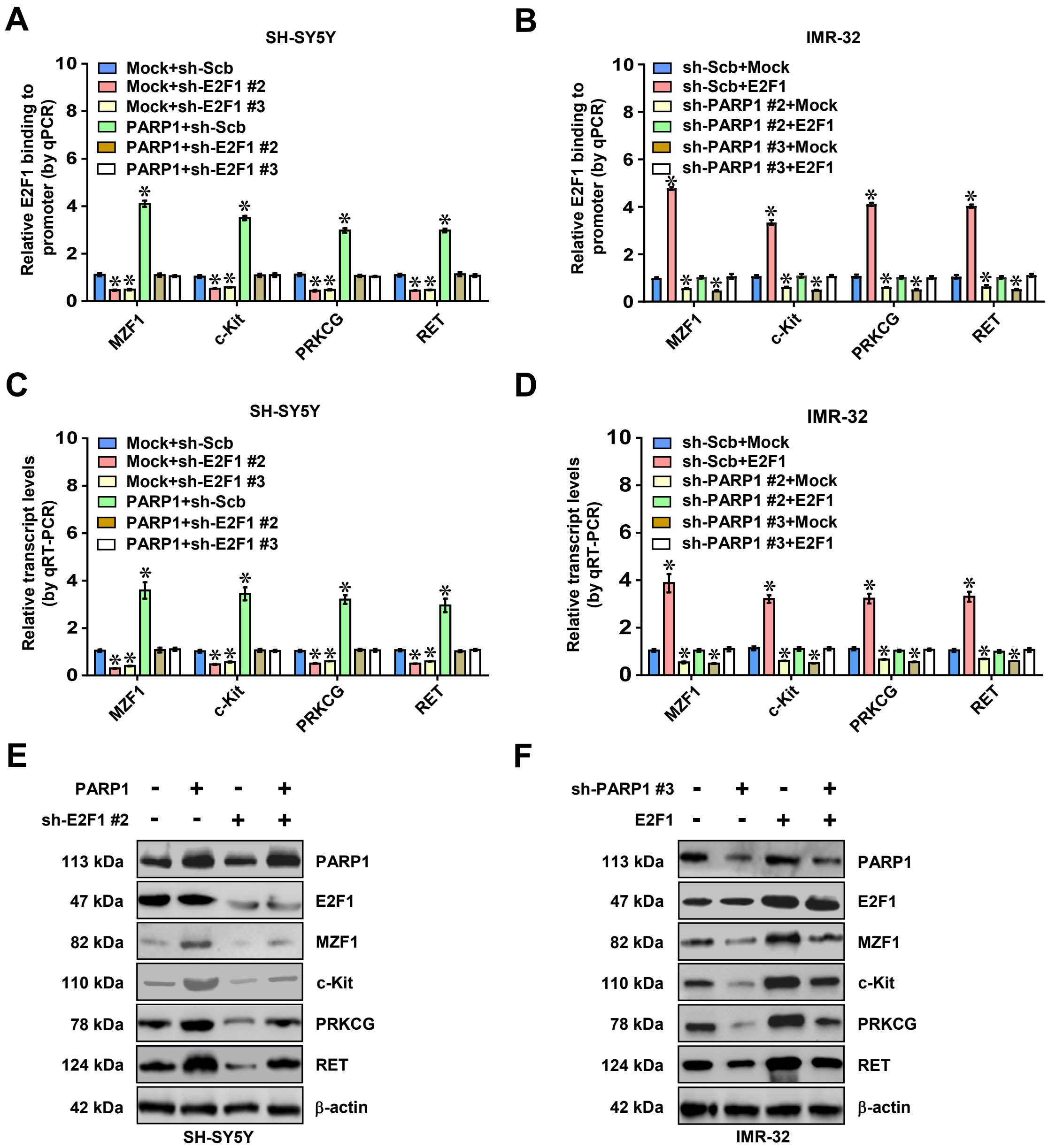

Figure S6. PARP1 regulates target gene expression through E2F1. A-D) ChIP and qPCR (A and B, normalized to input, *n*=4) and real-time qRT-PCR (C and D, normalized to β-actin, *n*=5) assays indicating the E2F1 enrichment and transcript levels of target genes in SH-SY5Y and IMR-32 cells stably transfected with empty vector (mock), *PARP1*, scramble shRNA (sh-Scb), or sh-PARP1, and those co-transfected with sh-E2F1 or *E2F1*. **E** and **F**) Western blot assay showing the protein levels of target genes in SH-SY5Y and IMR-32 cells stably transfected with mock, *PARP1*, sh-Scb, or sh-PARP1 #3, and those co-transfected with sh-E2F1 #2 or *E2F1*. Student's *t* test compared difference in **A-D**. * P<0.05 vs. mock+sh-Scb.

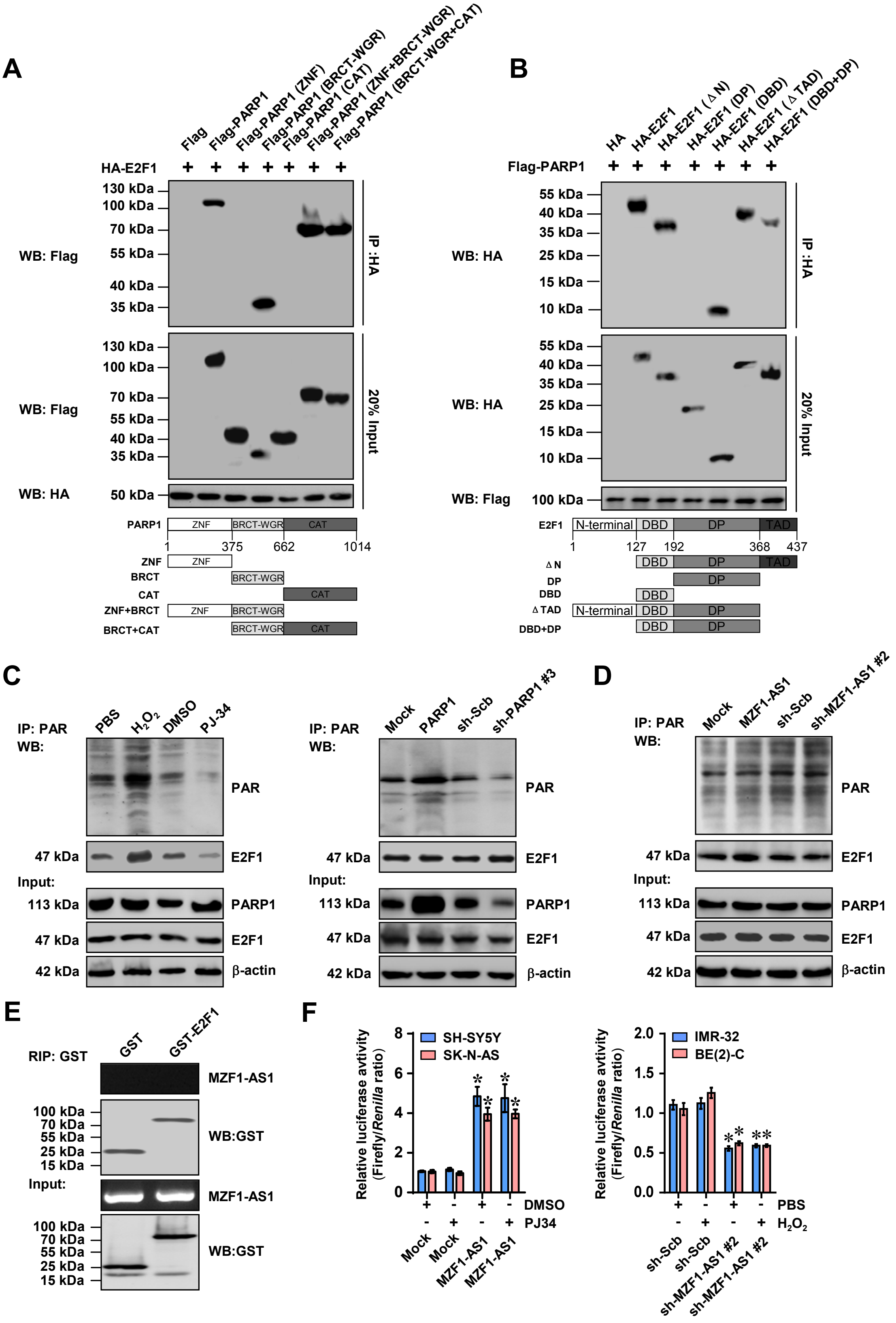

Figure S7. Interaction between PARP1 and E2F1 in NB cells. **A**) Co-IP and western blot assays (upper panel) indicating the interaction between PARP1 and E2F1 in SH-SY5Y cells transfected with full-length or truncations of Flag-tagged *PARP1* and HA-tagged *E2F1* as indicated (lower panel). **B**) Co-IP and western blot assays (upper panel) showing the interaction between PARP1 and E2F1 in SH-SY5Y cells transfected with full-length or truncations of HA-tagged *E2F1* and Flag-tagged *PARP1* as indicated (lower panel). **C**) Co-IP and western blot assays indicating the PARylation of E2F1 in lysates of SH-SY5Y cells treated with solvent controls, H_2O_2 (10 μmol/L), or PJ-34 (10 μmol/L), and those transfected with empty vector (mock), *PARP1*, scramble shRNA (sh-Scb), or sh-PARP1 #3. **D**) Co-IP and western blot assays showing the PARylation of E2F1 in lysates of SH-SY5Y cells transfected with mock, *MZF-1-AS1*, sh-Scb, or sh-MZF1-AS1 #2. **E**) RIP assay using GST antibody indicating the interaction between GST-tagged E2F1 recombinant protein and *MZF1-AS1* within IMR-32 cells. **F**) Dual-luciferase assay showing the activity of E2F1 in NB cells stably transfected with mock, *MZF1-AS1*, sh-Scb, or sh-MZF1-AS1#2, and those treated with solvent controls, PJ-34 (10 μmol/L), or H_2O_2 (10 μmol/L). Student's *t* test compared difference in **F**. * $P<0.05$ vs. mock+DMSO or sh-Scb+PBS. Data are shown as mean ± s.e.m. (error bars) and representative of three independent experiments in **A**-**F**.

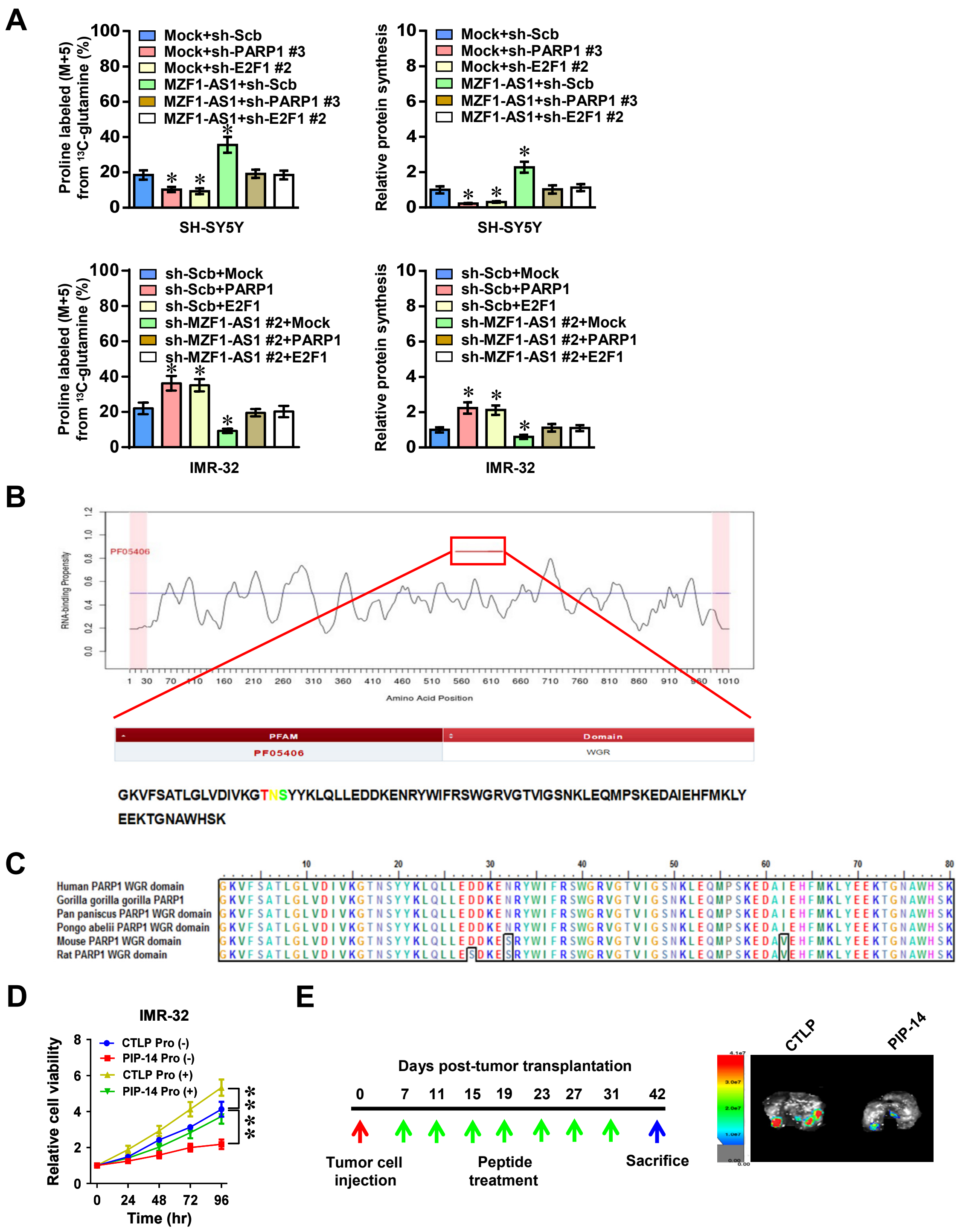

Figure S8. ***MZF1-AS1* facilitates proline synthesis and NB progression via PARP1 and E2F1. A**) LC-MS analysis of ^{13}C glutamine-to-proline flux and *de novo* global protein synthesis in SH-SY5Y and IMR-32 cells stably transfected with empty vector (mock), *MZF1-AS1*, scramble shRNA (sh-Scb), or sh-MZF1-AS1 #2, and those co-transfected with sh-PARP1 #3, sh-E2F1 #2, *PARP1*, or *E2F1* (*n*=5). **B** and **C**) Analysis using catRAPID program (B) and homologous analysis (C) of WGR domain of PARP1 protein revealing its potential roles in interacting with RNA. **D**) MTT colorimetric assay showing the viability of IMR-32 cells treated with mutant control peptide (CTLP) or PIP-14 (20 μmol/L), with or without proline (Pro, 3 mM) supplementation for time point as indicated (*n*=5). **E**) *In vivo* imaging of lungs (right panel) of nude mice (*n*=5 for each group) treated with tail vein injection of IMR-32 cells and subsequent administration of CTLP or PIP-14 (3 mg·kg^{-1}) as indicated (left panel). Student's *t* test compared difference in **A** and **D**. * P<0.05, **P<0.01 vs. mock+sh-Scb or CTLP Pro (-). Data are shown as mean ± s.e.m. (error bars) in **A** and **D**.

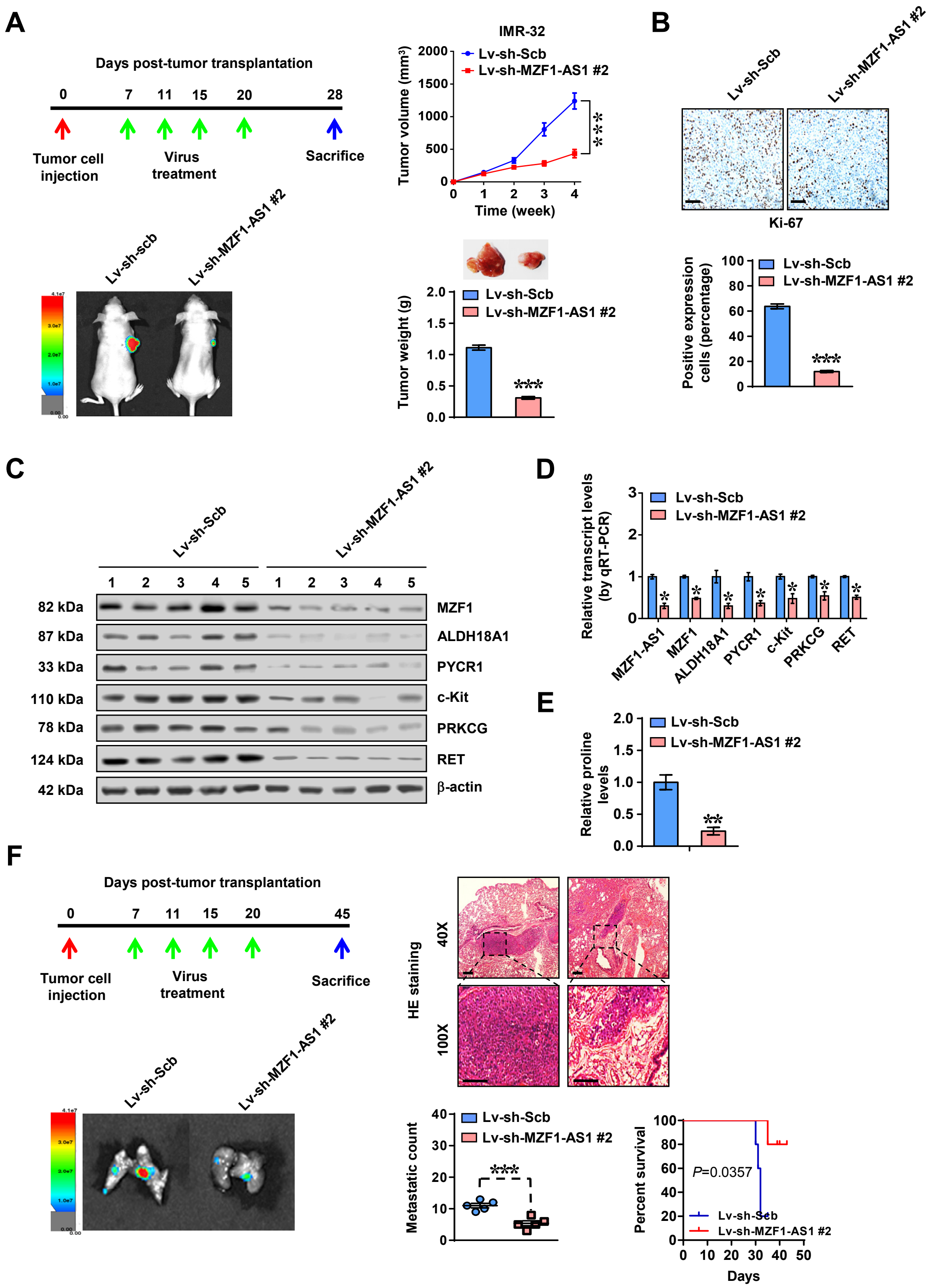

Figure S9. Lentivirus-mediated *MZF1-AS1* knockdown inhibits proline synthesis and NB progression. A) *In vivo* imaging (left lower panel), *in vivo* growth curve (right upper panel), representative images and weight at the end points (right lower panel) of xenograft tumors formed by subcutaneous injection of IMR-32 cells into dorsal flanks of athymic nude mice (n=5 per group) that received intratumoral injection of lentivirus (Lv)-mediated scramble shRNA (sh-Scb) or sh-MZF1-AS1 #2 as indicated (left upper panel). **B**) Representative images (upper panel) and quantification (lower panel) of immunohistochemical staining revealing Ki-67 expression within IMR-32 cells-formed subcutaneous xenograft tumors (n=5 per group) following intratumoral injection of lentivirus carrying sh-Scb or sh-MZF1-AS1 #2. Scale bars: 50 μm. **C** and **D**) Western blot (C) and real-time qRT-PCR (D, normalized to β-actin) assays showing the expression of *MZF1-AS1* and its target genes in xenograft tumors formed by subcutaneous injection of IMR-32 cells into dorsal flanks of athymic nude mice (n=5 per group) that received intratumoral injection of Lv-sh-Scb or Lv-sh-MZF1-AS1 #2. **E**) Proline levels in IMR-32 cells-formed xenograft tumors (n=5 per group) that received intratumoral injection of Lv-sh-Scb or Lv-sh-MZF1-AS1 #2. **F**) Lv-mediated therapy timeline (left upper panel), *in vivo* imaging of lungs (left lower panel), H&E staining images and metastatic counts of lungs (right upper and lower panels), and Kaplan-Meier curves (right lower panel) of nude mice (n=5 per group) treated with tail vein injection of IMR-32 cells and subsequent administration of Lv-mediated sh-Scb or sh-MZF1-AS1 #2. Scale bar: 100 μm. Student's t test and analysis of variance compared difference in **A**, **B** and **D-F**. Log-rank test for survival comparison in **F**. *P<0.05, ** P<0.01, *** P<0.001 vs. Lv-sh-Scb. Data are shown as mean ± s.e.m. (error bars).

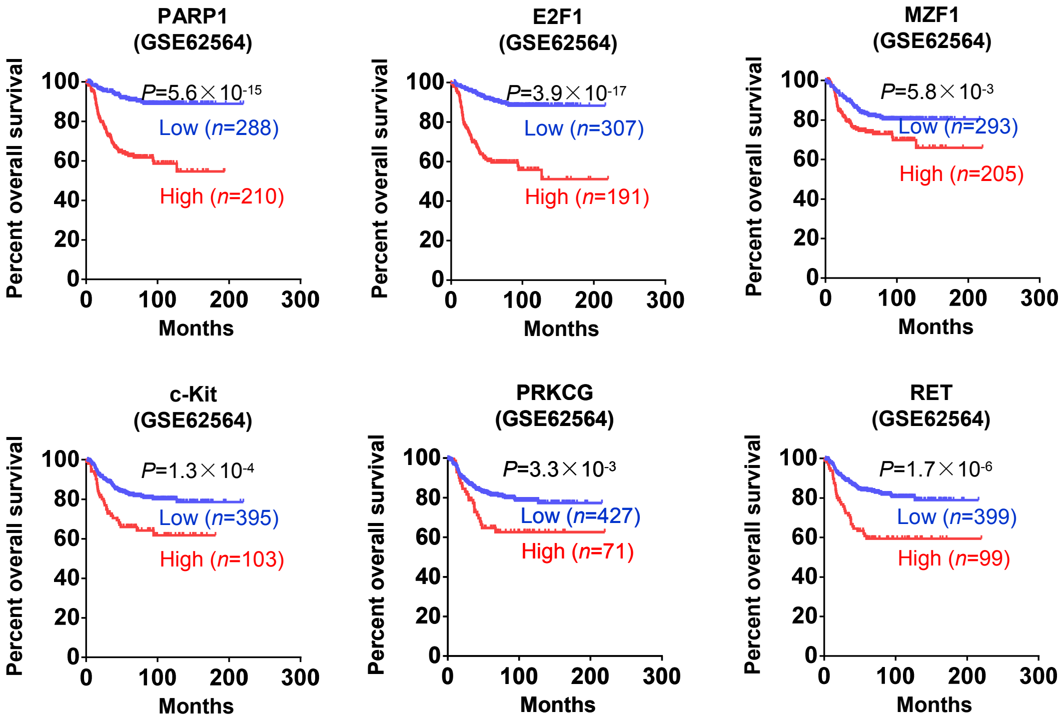

Figure S10. Kaplan-Meier survival plots of *PARP1*, *E2F1*, and target genes in a public NB dataset. Kaplan-Meier curves showing overall survival of 498 NB cases (GSE62564) with low or high expression levels of *PARP1* (cutoff value=139.717), *E2F1* (cutoff value=43.341), *MZF1* (cutoff value=35.2), *c-Kit* (cutoff value=10.145), *PRKCG* (cutoff value=1.4), or *RET* (cutoff value=24.2). Log-rank test for survival comparison.

Table S1 Mass spectrometry analysis of proteins pulled down by *MZF1-AS1*

ACAA1	DLAT	RFC3	UFD1L
ACP1	DPY30	RPS26P11	UGP2
ADGRL2	DRG2	RPS29	UHRF1
ADH1A	EHD4	RPS6KA3	VAC14
ANKFY1	EIF2S3	SAFB	VAMP2
ANXA6	EIF3B	SHMT1	VAMP3
AVIL	GSTM5	SLC25A6	ZADH2
C14orf1	HBG1	SLC27A3	ZMPSTE24
C16orf62	LSM12	SND1	ZW10
CAMK2B	MARS	SNR	
CAMK2D	MAT2A	SNRPGP15	
CBFB	NAA15	STEAP2	
CCAR2	NUP205	STK38	
CUL3	PARP1	SUPT16H	
CUL4A	PLOD1	TBL1Y	
DCAKD	PPP6C	THOP1	
DDX19B	PPP6R3	TWF1	
DHX30	PRRC1	TYMS	
DHX36	RBPJ	UBE2V1	
DHX37	RCN1	UBE2V2	

Table S2 WGR binding score

No.	Sequence	RNABindRPlus	Score	No.	Sequence	RNABindRPlus	Score
1	K	0	0.01	41	R	1	0.69
2	V	0	0.01	42	V	0	0.43
3	F	0	0.01	43	G	0	0.49
4	S	0	0.01	44	T	1	0.52
5	A	0	0.01	45	V	0	0.35
6	T	0	0.06	46	I	1	0.56
7	L	0	0.04	47	G	0	0.39
8	G	1	0.53	48	S	0	0.28
9	L	0	0.1	49	N	0	0.31
10	V	0	0.05	50	K	0	0.35
11	D	0	0.2	51	L	0	0.06
12	I	0	0.04	52	E	0	0.03
13	V	0	0.08	53	Q	0	0.04
14	K	1	0.56	54	M	0	0.02
15	G	0	0.21	55	P	0	0.02
16	T	1	0.66	56	S	0	0.04
17	N	1	0.6	57	K	0	0.04
18	S	1	0.66	58	E	0	0.01
19	Y	1	0.58	59	D	0	0.01
20	Y	0	0.46	60	A	0	0.01
21	K	0	0.03	61	I	0	0.01
22	L	0	0.02	62	E	0	0.01
23	Q	0	0.34	63	H	0	0.01
24	L	0	0.01	64	F	0	0.01
25	L	0	0.02	65	M	0	0.01
26	E	0	0.02	66	K	0	0.01
27	D	0	0.02	67	L	0	0.02
28	D	0	0.02	68	Y	0	0.02
29	K	0	0.07	69	E	0	0.04
30	E	0	0.06	70	E	0	0.02
31	N	0	0.17	71	K	0	0.45
32	R	0	0.08	72	T	1	0.54
33	Y	0	0.13	73	G	0	0.39
34	W	0	0.15	74	N	0	0.35
35	I	0	0.05	75	A	0	0.39
36	F	1	0.6	76	W	1	0.61
37	R	1	0.65	77	H	0	0.01
38	S	1	0.69	78	S	0	0.01
39	W	1	0.69	79	K	1	0.53
40	G	1	0.65				

Table S3 Primer sets used for qRT-PCR, RIP, RT-PCR, probe, and ChIP

Primer set	Primers	Sequence	Product size	Application
MZF1-AS1	Forward	5'-GGAATCTGCGTTTATGAAGGGGA-3'	197	qRT-PCR, RIP
	Reverse	5'-TTCTGGGAAGTGTATTTCGGGAC-3'		
MZF1-AS1 (Exon 1+2+3)	Forward	5'-CCCAAGCTTGTCACCTCCGCTGCTGCCGCCAC-3'	360	RT-PCR
	Reverse	5'-CCGGAATTCCTGAAACTGAGCCTCCAACTCCA-3'		
MZF1-AS1 (Exon 4+5)	Forward	5'-CCCAAGCTTGGATGGGATCTTACTATGTTGCCTA-3'	362	RT-PCR
	Reverse	5'-CCGGAATTCCTTGGCACCCTGGAGCGGAAGTTGC-3'		
MZF1-AS1 (Exon 6-1)	Forward	5'-CCCAAGCTTCCCTGGGCGCTCCCGCAAGGACT-3'	532	RT-PCR
	Reverse	5'-CCGGAATTCCTAATGACGGCCGTGATTGGTGC-3'		
MZF1-AS1 (Exon 6-2)	Forward	5'-CCCAAGCTTAACTCCGTCGGTGCCCCACCGCGT-3'	418	RT-PCR
	Reverse	5'-CCGGAATTCCCGCCGGTCCTTCGTCTCTCTTT-3'		
MZF1-AS1 (Exon 6-3)	Forward	5'-CCCAAGCTTGAGGAAGAGAGAGAGAGACAGAGA-3'	985	RT-PCR
	Reverse	5'-CCGGAATTCTGAGCATGTGTCAAAAGATTTATTTACCG-3'		
MZF1-AS1	Forward	5'-CTCAATCAGAATCTCCAGGAACGG-3'	185	Probe
	Reverse	5'-GTCACCTCCGCTGCTGCCGCCACC-3'		
GAPDH	Forward	5'-GGGCTGCTTTTAACTCTGGT-3'	198	qRT-PCR, Probe
	Reverse	5'-TGATTTTGGAGGGATCTCGC-3'		
U1	Forward	5'-ACTTACCTGGCAGGGGAGATACC-3'	137	qRT-PCR, Probe
	Reverse	5'-CCACTACCACAAATTATGCAGTCG-3'		
MZF1	Forward	5'-CTGAAACTGAGCCTCCAACTCC-3'	232	qRT-PCR,
	Reverse	5'-CCAGTCTTGCTGTGGGGAAA-3'		
HOTAIR	Forward	5'-AACTCTGACTCGCCTGTGCTCT-3'	233	RT-PCR
	Reverse	5'-CTTCTAAATCCGTTCCATTCCA-3'		
ALDH18A1	Forward	5'-GTGGTGACCCGAGGGGATGAA-3'	173	qRT-PCR
	Reverse	5'-CGCACGCTCTGAGACAGAAGG-3'		
ODC1	Forward	5'-GGGTGATTGGATGCTCTTTG-3'	164	qRT-PCR
	Reverse	5'-GTTCCTCTACTTCGGGTGGG-3'		
PYCR1	Forward	5'-GTCGTGGTGCGGGAGGGG-3'	174	qRT-PCR
	Reverse	5'-TGTGAATGCGTAGGCGGG-3'		
SLC7A5	Forward	5'-GGTGTACGTGCTGACCAACC-3'	176	qRT-PCR
	Reverse	5'-ATGTGAACAGGGACCCATTG-3'		
VARS	Forward	5'-TGGAAGGAGGAGAAAGGTGA-3'	168	qRT-PCR
	Reverse	5'-AAGGCGGGTACTGCGATAGA-3'		
PYCR2	Forward	5'-CATCCTGTCGGCTCACAAGATAATA-3'	124	qRT-PCR
	Reverse	5'-GTCGCTGTGCTTCACCGTCTCCTTG-3'		
PYCR3	Forward	5'-CTCATCAGAGCAGGAAAAGTGGAAG-3'	150	qRT-PCR
	Reverse	5'-GGTGGCAAAGATGACGAGCAGGCAG-3'		
PRODH	Forward	5'-ATCCAGCCCCTGCTTCGGCAC-3'	153	qRT-PCR
	Reverse	5'-CTTATTCGTGCCACTGCCATCCC-3'		
P5CDH	Forward	5'-GTGCAGTACCAAGTGTCGCCT-3'	127	qRT-PCR
	Reverse	5'-GCTTCAGGTCCCACTCTTTCCG-3'		
c-Kit	Forward	5'-GAAAAGGCAGAAGCCACCAACA-3'	124	qRT-PCR
	Reverse	5'-CATACAAGGAGCGGTCAACAAG-3'		
PRKCG	Forward	5'-GACGAAACAGAAGACCCGA-3'	119	qRT-PCR
	Reverse	5'-AGTCCCACACCTCCACGCT-3'		
RET	Forward	5'-GGACTGTAATAATGCCCCCC-3'	128	qRT-PCR
	Reverse	5'-TAGTGCCATCAGCTCTCGTG-3'		
E2F1	Forward	5'-AAGAACCGCTGTTGTCCCG-3'	182	qRT-PCR
	Reverse	5'-GAGGCCGAAGTGGTAGTCG-3'		
PARP1	Forward	5'-AAGGCGAATGCCAGCGTTAC-3'	190	qRT-PCR
	Reverse	5'-GCACTCTTGGAGACCATGTCA-3'		
β-actin	Forward	5'-TGCCCATCTACGAGGGGTATG-3'	156	qRT-PCR
	Reverse	5'-TCTCCTTAATGTCACGCACGATTT-3'		
ALDH18A1 (-197/+1)	Forward	5'-CAGGCACCTCTTCTACCTCCCT-3'	198	ChIP
	Reverse	5'-GTAGGCCCCTTTCGAGAATCAA-3'		
PYCR1 (-156/+14)	Forward	5'-CGCAGATGATGCAACCGCTC-3'	170	ChIP
	Reverse	5'-ACCCCAACCCAGCTACCGTG-3'		
MZF1 (-1598/-1450)	Forward	5'-ACGGAGTCTCGCTCTGTCG-3'	149	ChIP
	Reverse	5'-AAATTAGCCGGGCGTAGTG-3'		
c-Kit (+441/+638)	Forward	5'-GTGCGAGTTTGGGGTGGCTTT-3'	198	ChIP
	Reverse	5'-TCTCTAACTTTGCCCTCAGCG-3'		
RET (-546/-370)	Forward	5'-CGCTCCCACTGCGCCTCTCG-3'	177	ChIP
	Reverse	5'-CCCTGCCTTTTGCCCTTTCC-3'		
PRKCG (-198/-15)	Forward	5'-GCGGGGCAGGATGACAGAT-3'	184	ChIP
	Reverse	5'-CCAGGACGACCGAGAGGGC-3'		

MZF1-AS1, myeloid zinc finger 1 antisense RNA 1; GAPDH, glyceraldehyde 3-phosphate dehydrogenase; MZF1, myeloid zinc finger 1; HOTAIR, HOX transcript antisense RNA; ALDH18A1, aldehyde dehydrogenase 18 family member A1; ODC1, ornithine decarboxylase 1; PYCR, pyrroline-5-carboxylate reductase; SLC7A5, solute carrier family 7 member 5; VARS, valyl-tRNA synthetase; PRODH, proline dehydrogenase 1; P5CDH, P5C dehydrogenase; RET, ret proto-oncogene; PRKCG, protein kinase C gamma; E2F1, E2F transcription factor 1; PARP1, poly(ADP-ribose) polymerase 1.

Table S4 Primer sets used for constructs

Primer set	Primer	Sequence
pGL3-ALDH18A1 Luc (-281/+225)	Forward	5'-CGGGGTACCAACCTATTTCCGAGGTGTGCCCGGG-3'
	Reverse	5'-CCCAAGCTTTCGCCGCGTACCTTCCTCACCACCG-3'
pGL3-PYCR1 Luc (-471/+106)	Forward	5'-CGGGGTACCCCGGGCTTCTCCAAACTCGATGACA-3'
	Reverse	5'-GCCGCTCGAGTAGGGGTCCCCCGTCTGGGTTCCCA-3'
pcDNA3.1-MZF1-AS1	Forward	5'-CCCAAGCTTATCTTAAGTTGTATATATGTGCA-3'
	Reverse	5'-CCGGAATTCTGAGCATGTGTCAAAAGATTTATTTACCG-3'
pcDNA3.1-MZF1-AS1 (Exon 1+2+3)	Forward	5'-CCCAAGCTTATCTTAAGTTGTATATATGTGCA-3'
	Reverse	5'-CCGGAATTCCTGAAACTGAGCCTCCAACTCCA-3'
pcDNA3.1-MZF1-AS1 (Exon 4+5)	Forward	5'-CCCAAGCTTGGATGGGATCTTACTATGTTGCCTA-3'
	Reverse	5'-CCGGAATTCCTTGGCACCCTGGAGCGGAAGTTGC-3'
pcDNA3.1-MZF1-AS1 (Exon 6-1)	Forward	5'-CCCAAGCTTCCCTGGGCGCTCCCGCAAGGACT-3'
	Reverse	5'-CCGGAATTCCTAATGACGGCCGTGATTGGTGC-3'
pcDNA3.1-MZF1-AS1 (Exon 6-2)	Forward	5'-CCCAAGCTTAACTCCGTCGGTGCCCCACCGCGT-3'
	Reverse	5'-CCGGAATTCCCGCCGGTCCTTCGTCTCTCTTT-3'
pcDNA3.1-MZF1-AS1 (Exon 6-3)	Forward	5'-CCCAAGCTTGAGGAAGAGAGAGAGAGACAGAGA-3'
	Reverse	5'-CCGGAATTCTGAGCATGTGTCAAAAGATTTATTTACCG-3'
p3XFlag-CMV-10-PARP1-Full	Forward	5'-CGGAAGATCTGATGGCGGAGTCTTCGGATAAGCTCT-3'
	Reverse	5'-CTAGTCTAGATTACCACAGGGAGGTCTTAAAATTG-3'
p3XFlag-CMV-10-PARP1-ZFN	Forward	5'-CGGAAGATCTGATGGCGGAGTCTTCGGATAAGCTCT-3'
	Reverse	5'-CTAGTCTAGACGAGGCTGTGGAGGGCGGAGGCGT-3'
p3XFlag-CMV-10-PARP1-BRCT	Forward	5'-CGGAAGATCTGGCTCCTGCTGCTGTGAACTCCTCTG-3'
	Reverse	5'-CTAGTCTAGACTTGGTGCCAGGATTTACTGTCAGC-3'
p3XFlag-CMV-10-PARP1-CAT	Forward	5'-CGGAAGATCTGTCCAAGCTCCCCAAGCCAGTTCAGG-3'
	Reverse	5'-CTAGTCTAGATTACCACAGGGAGGTCTTAAAATTG-3'
p3XFlag-CMV-10-PARP1-ZFN+BRCT	Forward	5'-CGGAAGATCTGATGGCGGAGTCTTCGGATAAGCTCT-3'
	Reverse	5'-CTAGTCTAGACTTGGTGCCAGGATTTACTGTCAGC-3'
p3XFlag-CMV-10-PARP1-BRCT+CAT	Forward	5'-CGGAAGATCTGGCTCCTGCTGCTGTGAACTCCTCTG-3'
	Reverse	5'-CTAGTCTAGACTTGGTGCCAGGATTTACTGTCAGC-3'
pGEX-6P-1-PARP1-Full	Forward	5'-TCCCCCCGGGGATGGCGGAGTCTTCGGATAAGCTCT-3'
	Reverse	5'-GCCGCTCGAGTTACCACAGGGAGGTCTTAAAATTG-3'
pGEX-6P-1-PARP1-ZFN	Forward	5'-TCCCCCCGGGGATGGCGGAGTCTTCGGATAAGCTCT-3'
	Reverse	5'-GCCGCTCGAGCGAGGCTGTGGAGGGCGGAGGCGT-3'
pGEX-6P-1-PARP1-BRCT	Forward	5'-TCCCCCCGGGGGCTCCTGCTGCTGTGAACTCCTCTG-3'
	Reverse	5'-GCCGCTCGAGCTTGGTGCCAGGATTTACTGTCAGC-3'
pGEX-6P-1-PARP1-CAT	Forward	5'-TCCCCCCGGGGTCCAAGCTCCCCAAGCCAGTTCAGG-3'
	Reverse	5'-GCCGCTCGAGTTACCACAGGGAGGTCTTAAAATTG-3'
pGEX-6P-1-PARP1-ZFN+BRCT	Forward	5'-TCCCCCCGGGGATGGCGGAGTCTTCGGATAAGCTCT-3'
	Reverse	5'-GCCGCTCGAGCTTGGTGCCAGGATTTACTGTCAGC-3'
pGEX-6P-1-PARP1-BRCT+CAT	Forward	5'-TCCCCCCGGGGGCTCCTGCTGCTGTGAACTCCTCTG-3'
	Reverse	5'-GCCGCTCGAGTTACCACAGGGAGGTCTTAAAATTG-3'
PARP1 WGR Domain Mut	Forward	5'-CGTTAAAGGATCCTACGCCTACTACAAGCTGCAGCTTCTGGAGGACGA-3'
	Reverse	5'-CTTGTAGTAGGCGTAGGATCCTTTAACGATGTCCACCAGGCCAAGGGT-3'
pGL3-E2F1 Luc	Forward	5'-CTTTGGCGCATATTTCGCGCTATTTTCCCGCATATTTGCCGCA-3'
	Reverse	5'-AGCTTGCGGCAAATATGCGGGAAAATAGCGCGAAATATGCGCCAAAGG TAC-3'
pCMV-HA-E2F1-Full	Forward	5'-CCGGAATTCGGATGGCCTTGGCCGGGGCCCCTGCGGGCGGCCCAT-3'
	Reverse	5'-CGGGGTACCTCAGAAATCCAGGGGGGTGAG-3'
pCMV-HA-E2F1-ΔN-terminal	Forward	5'-CCGGAATTCGGTATGAGACCTCACTGAATCTG-3'
	Reverse	5'-CGGGGTACCTCAGAAATCCAGGGGGGTGAG-3'
pCMV-HA-E2F1-ΔTAD	Forward	5'-CCGGAATTCGGATGGCCTTGGCCGGGGCCCCTGCGGGCGGCCCAT-3'
	Reverse	5'-CGGGGTACCGGGAGCCCGCAGGCTGCCCAT-3'
pCMV-HA-E2F1-DBD	Forward	5'-CCGGAATTCGGTATGAGACCTCACTGAATCTG-3'
	Reverse	5'-CGGGGTACCGCCCAGCCACTGGATGTGGTT-3'
pCMV-HA-E2F1-DP	Forward	5'-CCGGAATTCGGAGCCACACCACAGTGGGCGTCGGC-3'
	Reverse	5'-CGGGGTACCGGGAGCCCGCAGGCTGCCCAT-3'
pCMV-HA-E2F1-DBD + DP	Forward	5'-CCGGAATTCGGTATGAGACCTCACTGAATCTG-3'
	Reverse	5'-CGGGGTACCGGGAGCCCGCAGGCTGCCCAT-3'

ALDH18A1, aldehyde dehydrogenase 18 family member A1; PYCR1, pyrroline-5-carboxylate reductase 1; MZF1-AS1, myeloid zinc finger 1 antisense RNA 1; PARP1, poly(ADP-ribose) polymerase 1; E2F1, E2F transcription factor 1.

Table S5 Oligonucleotides encoding short hairpin RNAs

Oligo Set	Sequences	
sh-Scb	5'-CCGGGCGAACGATCGAGTAAACGGACTCGAGTCCGTTTACTCGATCGTTCGCTTTTT-3'	(sense);
	5'-AATTCAAAAAGCGAACGATCGAGTAAACGGACTCGAGTCCGTTTACTCGATCGTTCGC-3'	(antisense)
sh-MZF1 #1	5'-CCGGCCGGCCTGCCTTTGACCCTATCTCGAGATAGGGTCAAAGGCAGGCCGGTTTTTG-3'	(sense);
	5'-GATCCAAAAACCGGCCTGCCTTTGACCCTATCTCGAGATAGGGTCAAAGGCAGGCCGG-3'	(antisense)
sh-MZF1 #2	5'-CCGGGACTGGGAGAGAATGCCCAGTCTCGAGACTGGGCATTCTCTCCCAGTCTTTTTG-3'	(sense);
	5'-GATCCAAAAAGACTGGGAGAGAATGCCCAGTCTCGAGACTGGGCATTCTCTCCCAGTC-3'	(antisense)
sh-MZF1-AS1 #1	5'-CCGGCCGGCCTGCCTTTGACCCTATCTCGAGATAGGGTCAAAGGCAGGCCGGTTTTTG-3'	(sense);
	5'-GATCCAAAAACCGGCCTGCCTTTGACCCTATCTCGAGATAGGGTCAAAGGCAGGCCGG-3'	(antisense)
sh-MZF1-AS1 #2	5'-CCGGGACTGGGAGAGAATGCCCAGTCTCGAGACTGGGCATTCTCTCCCAGTCTTTTTG-3'	(sense);
	5'-GATCCAAAAAGACTGGGAGAGAATGCCCAGTCTCGAGACTGGGCATTCTCTCCCAGTC-3'	(antisense)
sh-ALDH18A1 #1	5'-CCGGTGCCTTCAGTCATCAGACATGTCTCGAGACATGTCTGATGACTGAAGGCTTTTTG-3'	(sense);
	5'-GATCCAAAAAGCCTTCAGTCATCAGACATGTCTCGAGACATGTCTGATGACTGAAGGCA-3'	(antisense)
sh-ALDH18A1 #2	5'-CCGGTGCACGTCATCACAGACATTGTCTCGAGACAATGTCTGTGATGACGTGCTTTTTG-3'	(sense);
	5'-GATCCAAAAAGCACGTCATCACAGACATTGTCTCGAGACAATGTCTGTGATGACGTGCA-3'	(antisense)
sh-PYCR1 #1	5'-CCGGTGAAGCTGTCAGCGTTTCGGCTCGAGCCGAAACGCTGACAGCTTCTTTTTG-3'	(sense);
	5'-GATCCAAAAAGAAGCTGTCAGCGTTTCGGCTCGAGCCGAAACGCTGACAGCTTCA-3'	(antisense)
sh-PYCR1 #2	5'-CCGGTTAGGCGCCGACATTGAGGACTCGAGTCCTCAATGTCGGCGCCTATTTTTG-3'	(sense);
	5'-GATCCAAAAATAGGCGCCGACATTGAGGACTCGAGTCCTCAATGTCGGCGCCTAA-3'	(antisense)
sh-PARP1 #1	5'-CCGGGCAGCTTCATAACCGAAGATTCTCGAGAATCTTCGGTTATGAAGCTGCTTTTTG-3'	(sense);
	5'-GATCCAAAAAGCAGCTTCATAACCGAAGATTCTCGAGAATCTTCGGTTATGAAGCTGC-3'	(antisense)
sh-PARP1 #2	5'-CCGGCCGAGAAATCTCTTACCTCAACTCGAGTTGAGGTAAGAGATTTCTCGGTTTTTG-3'	(sense);
	5'-GATCCAAAAACCGAGAAATCTCTTACCTCAACTCGAGTTGAGGTAAGAGATTTCTCGG-3'	(antisense)
sh-PARP1 #3	5'-CCGGGCTTCACATATCAGCAGGTTACTCGAGTAACCTGCTGATATGTGAAGCTTTTTG-3'	(sense);
	5'-GATCCAAAAAGCTTCACATATCAGCAGGTTACTCGAGTAACCTGCTGATATGTGAAGC-3'	(antisense)
sh-E2F1 #1	5'-CCGGCACTGACTCTGCCACCATAGTCTCGAGACTATGGTGGCAGAGTCAGTGTTTTTG-3'	(sense);
	5'-GATCCAAAAACACTGACTCTGCCACCATAGTCTCGAGACTATGGTGGCAGAGTCAGTG-3'	(antisense)
sh-E2F1 #2	5'-CCGGACCTCTTCGACTGTGACTTTGCTCGAGCAAAGTCACAGTCGAAGAGGTTTTTTG-3'	(sense);
	5'-GATCCAAAAAACCTCTTCGACTGTGACTTTGCTCGAGCAAAGTCACAGTCGAAGAGGT-3'	(antisense)
sh-E2F1 #3	5'-CCGGGACCTCTTCGACTGTGACTTTCTCGAGAAAGTCACAGTCGAAGAGGTCTTTTTG-3'	(sense);
	5'-GATCCAAAAAGACCTCTTCGACTGTGACTTTCTCGAGAAAGTCACAGTCGAAGAGGTC-3'	(antisense)

MZF1, myeloid zinc finger 1; MZF1-AS1, myeloid zinc finger 1 antisense RNA 1; ALDH18A1, aldehyde dehydrogenase 18 family member A1; PYCR1, pyrroline-5-carboxylate reductase 1; PARP1, poly(ADP-ribose) polymerase 1; E2F1, E2F transcription factor 1.
